# The McKenna reaction – avoiding side reactions in phosphonate deprotection

**DOI:** 10.3762/bjoc.16.119

**Published:** 2020-06-23

**Authors:** Katarzyna Justyna, Joanna Małolepsza, Damian Kusy, Waldemar Maniukiewicz, Katarzyna M Błażewska

**Affiliations:** 1 Institute of Organic Chemistry, Faculty of Chemistry, Lodz University of Technology, Zeromskiego St. 116, 90-924 Lodz, Poland; 2 Institute of General and Ecological Chemistry, Faculty of Chemistry, Lodz University of Technology, Zeromskiego St. 116, 90-924 Lodz, Poland

**Keywords:** bromotrimethylsilane, McKenna reaction, organophosphorus acid, oxazole, phosphonate ester

## Abstract

The McKenna reaction is a well-known and popular method for the efficient and mild synthesis of organophosphorus acids. Bromotrimethylsilane (BTMS) is the main reagent in this reaction, which transforms dialkyl phosphonate esters into bis(trimethylsilyl)esters, which are then easily converted into the target acids. However, the versatile character of the McKenna reaction is not always used to its full extent, due to formation of side products. Herein, demonstrated by using model examples we have not only analyzed the typical side processes accompanying the McKenna reaction, but also uncovered new ones. Further, we discovered that some commonly recommended precautions did not always circumvent the side reactions. The proposed results and recommendations may facilitate the synthesis of phosphonic acids.

## Introduction

The McKenna reaction is a tool for the synthesis of organophosphorus acids from their esters and known for over 40 years [1–2]. The importance of this class of compounds is widely recognized as phosphorus acids and esters are prevailing in nature [3–4], and the compounds found wide applications as therapeutics [5–6], probes [7] or in materials science [8].

**Scheme 1:**
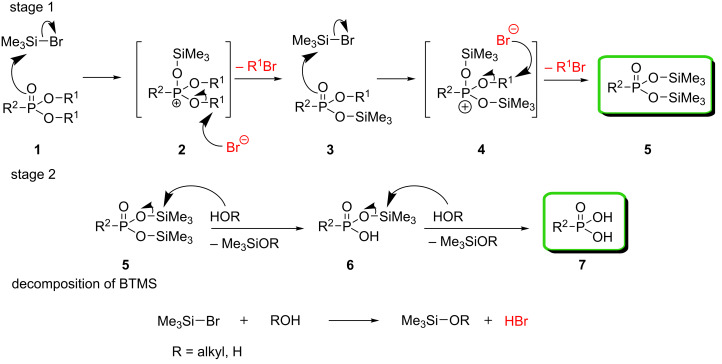
Schematic overview of the McKenna reaction including the decomposition of BTMS in protic solvents. The desired products of the McKenna reaction are presented in green and the reagents that potentially are responsible for side reactions are presented in red.

The McKenna reaction involves two steps: In the first step an alkyl ester is transformed into the corresponding trimethylsilyl ester [9], which is cleaved in the second step, upon solvolysis, forming the final product (Scheme 1). Bromotrimethylsilane (BTMS) is the main reagent in this reaction and is also known for its ability to cleave lactones, epoxides, acetals, and ethers [10]. BTMS also acted as a brominating agent and reagent for the formation of silyl enol ethers [10]. However, these reactions often required higher temperatures (up to 100 °C) or were applicable to only certain types of functional groups, such as methoxymethyl ethers [10]. Still, BTMS, due to its balanced effectiveness and high chemoselectivity, is the reagent of choice for phosphonate ester cleavage, compared with its more and less reactive analogs, iodotrimethylsilane (ITMS) [11–12] and chlorotrimethylsilane (CTMS) [13], respectively.

While being one of the most popular methods for the deprotection of organophosphorus esters, the McKenna reaction may be accompanied by side reactions such as the cleavage of *tert*-butyl carboxyester, [14–15] or other ester groups [16], as well as the formation of decomposition products [17–20]. Instead of focusing only on the experiments that “did work”, we decided to follow an alternative strategy recommended by Björnmalm and Caruso [21] and to report failures, as a tool to facilitate and accelerate new studies requiring the synthesis of phosphonic acids. To the best of our knowledge, this is the first systematic study on side reactions accompanying the McKenna reaction.

It is important to distinguish the side reactions, which originate from BTMS itself, and those resulting from not having taken appropriate precautions during the subsequent solvolysis step. By being alerted to the water-sensitive character of BTMS and the formation of the alkylating agent alkyl bromide (Scheme 1), side reactions occurring during the silylation step could be usually prevented. On the other hand, the side reactions during the solvolysis step are usually obviated by the use of a buffer or weak base, which neutralize the final organophosphorus acids. Herein we mainly addressed the problems related to the silylation step of the McKenna reaction. To this end, we used model compounds from our previous work, analyzed the possible side reactions, and proposed solutions in order to minimize or prevent the unwanted processes. We also showed that tertiary amines, that are commonly used as additives to prevent these side reactions, should be used with caution, as they themselves may promote side reactions.

## Results and Discussion

Our objective was to subject selected bifunctional compounds to the McKenna reaction conditions. In order to simplify the design of the study, in most cases the reaction mixture contained two model compounds, with one representing the phosphonate, and the other containing a functional group, which could undergo a side reaction under the reaction conditions.

**Figure 1:**
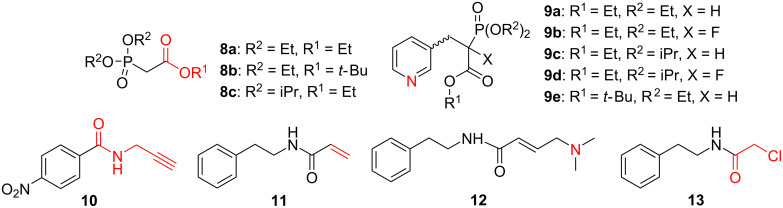
The model compounds used for this study (in red: the functionality of the molecules vulnerable to side reactions).

As the first class of compounds, representing the phosphoryl group, we used phosphonates **8** and **9** (Figure 1). As the second class of model compounds, which should remain intact under McKenna’s reaction conditions, we synthesized analogs **9**, and **10–13** (Figure 1). Among the representative functional groups, we selected compounds comprising a triple (**10**) and a double (**11**) bond, both susceptible to HBr addition. Compounds **12** and **9a–d** were selected for studying the possibility of an *N*-alkylation by alkyl bromide, formed during the silylation step. Finally, compound **13** was included to study the possibility of a chlorine for bromine exchange reaction. The phosphonocarboxylates **8** and **9** were used to explore whether the carboxyester group could be cleaved upon exposure to BTMS. All model compounds were prepared according to literature procedures and thoroughly dried before use [22–25].

Even though the McKenna reaction could be completed within a couple of hours, the rate of the reaction depends on the structure of the phosphonate ester, being especially affected by the type of substituents in the vicinity of the phosphonic group [1]. Usually the reaction is carried out for 1–3 days [26–27], in a solution of DCM or acetonitrile (ACN), although it can also be performed in neat BTMS. As far as the reaction temperature is concerned it can be performed either at room temperature, in refluxing CH_2_Cl_2_ [19,28–29], at higher temperatures [1,30–31], and also under microwave conditions [32–34]. At lower temperatures or shorter reaction times an incomplete transesterification was reported [35]. The use of the low-boiling CH_2_Cl_2_ at reflux promotes the removal of EtBr from the reaction mixture.

For the purpose of this study, we used the polar aprotic solvent acetonitrile due to its good solubilizing properties. The reactions were usually carried out at 35 °C for 24 h, based on our experience with different types of phosphonate esters that indicated a more efficient transesterification reaction at this temperature. Depending on the experiment, the progress of the reaction was monitored by ^1^H and ^31^P NMR spectroscopy in short intervals. The complete deprotection of the phosphonate ester group in compound **8** and **9** was observed.

In all experiments, unless specified otherwise, we used commercially available BTMS, which was distilled prior to use, kept in ampules under Ar, and stored frozen in the dark. These precautions were necessary in order to eliminate the concern that the studied side reactions were due to the presence of HBr in the commercially available reagent.

We divided the study into five sections, each devoted to a different side reaction, summarizing the applied conditions, identifying the culprit responsible for a particular side reaction and indicating the optimal conditions, which helped to circumvent the problem.

### Section 1: Formation of oxazoles from propargylamides

One of the most intriguing side reactions was observed when compounds comprising a propargyl amide group were treated with BTMS. Besides the products of addition of HBr, we observed the formation of oxazole.

**Scheme 2:**
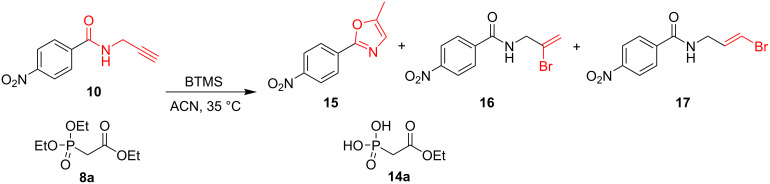
Formation of the side products derived from **10**. Conditions: An equimolar mixture of propargylamide **10** and triethyl phosphonoacetate (**8a**) in ACN stirred at 35 °C in the presence of BTMS.

In order to study this process, we subjected an equimolar mixture of the easily available propargylamide **10** [22] and triethyl phosphonoacetate (**8a**) to BTMS (Scheme 2). Besides the target product of the McKenna reaction, phosphonic acid **14a**, we also isolated a mixture of three compounds **15–17**, derived from propargylamide **10**, the compound, which should have remained intact under the conditions applied (Table 1, entry 1). The disappearance of the alkyne proton signals in the ^1^H NMR spectrum (δ 2.32 ppm (t, ^4^*J*_HH_ = 2.6 Hz) and δ 4.28 ppm (dd, ^3^*J*_HH_ = 5.2 Hz; ^4^*J*_HH_ = 2.5 Hz)) and, instead, the appearance of signals at δ 2.44 ppm (d, ^4^*J*_HH_ = 3.5 Hz) and δ 6.94 ppm (q, ^4^*J*_HH_ = 3.5 Hz) indicated the formation of oxazole **15**. The presence of compound **15** was also confirmed by X-ray studies (see Figure S3 and Table S1 in Supporting Information File 1) and literature data [36]. Additionally, the formation of compounds **16** and **17** was observed, resulting from HBr addition to the triple bond. The presence of these products was confirmed by ^1^H NMR analysis [signals at δ 5.56 and δ 5.85 ppm representing protons in **16** (CBr=C*H*_2_) and multiplets at δ 6.29 and 6.33 ppm representing protons in **17** (C*H*=C*H*Br)].

**Table 1:** Investigation of the reaction between propargylamide **10** and BTMS.

entry	BTMS^a^	TEA	other (equiv)	time [h]	ratio **10**:**15**:**16**:**17**

1^b^	+	–	–	24	0.78:0.07:0.07:0.08
2	+^c^	–	Cu	24	0.39:0.23:0.20:0.18
3	+	–	CuBr (0.5)	48	0.68:0.07:0.09:0.07^d^
4^e^	–	–	CuBr (0.1)	48	1:0:0:0
5	+	–	H_2_O (2)	24	0:0.63:0.2:0.17
**6**	**+**	**1 equiv**	**–**	**24**	**1:0:0:0**

7	–	–	33% HBr in AcOH (1)	24	0.67:0.27:0.03:0.03
8	–	–	TFA (1.3)	24	1:0:0:0

^a^BTMS (12 equiv) distilled and stored under Ar in a sealed ampule was used, unless otherwise stated; ^b^the same result was obtained when the reaction was carried out in a Schlenk apparatus, under strictly anhydrous conditions; ^c^commercially available reagent stabilized with copper wire was used; ^d^the dibrominated product derived from **10** was also observed (9%); ^e^the reaction was carried out in the absence of triethyl phosphonoacetate (**8a**).

In order to identify the factor(s) responsible for the formation of product **15**, we investigated whether the copper wire, used as a stabilizer in commercially available BTMS, could induce the observed cyclization, as was reported in the literature [37]. For that purpose, we compared the reaction run using commercially available BTMS, stabilized with copper wire with that using BTMS distilled prior to use but contaminated with CuBr (Table 1, entries 2 and 3). In both cases the byproducts **15**–**17** formed. However, when propargylamide **10** was subjected to the reaction with CuBr, in the absence of BTMS and phosphonoacetate, only starting material was recovered (Table 1, entry 4).

Next, we studied whether the presence of water in the reaction mixture could be responsible for the generation of the side products **15**–**17**. It has been reported previously that hydrogen bromide generated in situ by the addition of equimolar amounts of BTMS and MeOH to a solution of acetylenic ethers gave α-halovinyl ethers [38]. HBr generated in situ from BTMS and water was also used in the synthesis of α-bromoenamides from an ynamide [39]. However, to the best of our knowledge, there are no reports on the formation of oxazoles in this manner [40].

We found that the addition of water (2 equiv) to the reaction mixture with BTMS (12 equiv, Table 1, entry 5) resulted in the complete conversion of substrate **10** into compounds **15**–**17** within 24 h. In this case, over 60% of the reaction mixture constituted compound **15**, while the regioisomers **16** and **17** were formed in comparable amounts. Decreasing the amount of water to 0.5 equiv or lowering the temperature (from 35 °C to ambient temperature) decelerated the side reaction.

Unfortunately, even when carrying out the reaction under strictly anhydrous conditions (Table 1, entry 1), a 22% conversion of the substrate into the side products **15–17** was observed. Therefore, we repeated the reaction with triethylamine (TEA) added to the reaction mixture. These conditions appeared very promising, as no side products were formed and only the desired product **14a** was obtained (Table 1, entry 6).

To address the question whether compounds **16** and **17** constitute intermediates in the reaction leading to **15**, we isolated the mixture of **16** and **17**. The exposure of this mixture to BTMS, in the presence or absence of water, left them unaffected, indicating that the formation of the side products **15** and **16**,**17** proceeded through independent pathways.

Since the known procedures for the syntheses of oxazoles are rather harsh and usually involve higher temperature [Pd(OAc)_2_, toluene, AcOH, 100 °C, 24 h [41] and/or the presence of Lewis acids (FeCl_3_, ACN (24 h, 80 °C) or 1,2-DCE (2 h, 80 °C), DCM (24 h, 45 °C) [40]], we envisioned that the present method could be of synthetic value, if we were able to redirect the reaction towards the exclusive formation of oxazole **15**. However, our attempts at optimizing the conditions towards product **15** only led to partial success (see Table S2 in Supporting Information File 1) and the highest conversion of **10** into **15** was 63%. Still, even though the transformation was not fully selective, the pure oxazole **15** could be easily isolated from the reaction mixture in good yield (>50%). The products **15**–**17** did also form when compound **10** was treated with HBr in AcOH (Table 1, entry 7). However, all attempts at eliminating the formation of the addition products **16** and **17**, by using other acids (e.g., TFA, AcOH, HCl, or CF_3_SO_3_H) failed and only substrate **10** was recovered (see Table 1, entry 8, and Supporting Information File 1, Table S2).

### Section 2: Addition of HBr to the double bond

**Scheme 3:**
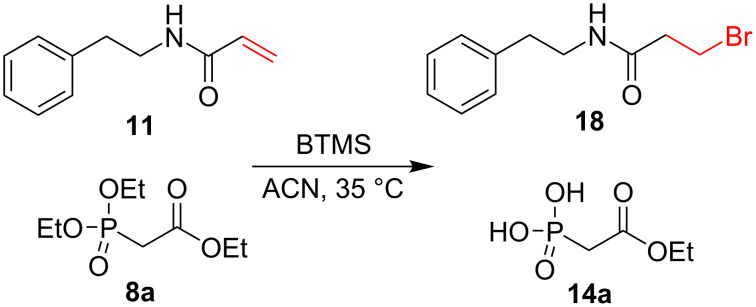
Addition of HBr to compound **11**.

HBr might form upon careless storage and/or use of BTMS, which exposes it to traces of water. As mentioned in the previous section, compounds having multiple bonds could undergo an addition reaction upon exposure to HBr [42]. We studied this problem using ^1^H NMR spectroscopy with the model acrylamide **11** (Scheme 3), representing a Michael acceptor, as such is currently popular in the development of covalent inhibitors [43]. The reaction was carried out in the presence of 12 equiv BTMS at 35 °C. The disappearance of the signals originating from the vinyl protons (multiplets at δ 5.56–5.66 and 5.95–6.10 ppm) and the appearance of the signals corresponding to the CH_2_CH_2_Br group in **18** (multiplets at δ 2.69 and 3.61 ppm) were taken as indicators of the side reaction. We found that the product of HBr addition formed only upon the intentional addition of water, due to the formation of HBr from BTMS, while under standard anhydrous conditions no side product was observed. The reaction was stoichiometric and in the case of adding 0.5 equiv of water, the conversion into **18** reached 47% within 24 h at 35 °C. The addition of TEA (1 equiv) protected compound **11** against HBr addition, as we have observed in the experiment with 0.5 equiv of water and 1 equiv of TEA.

### Section 3: *N*-Alkylation

**Scheme 4:**
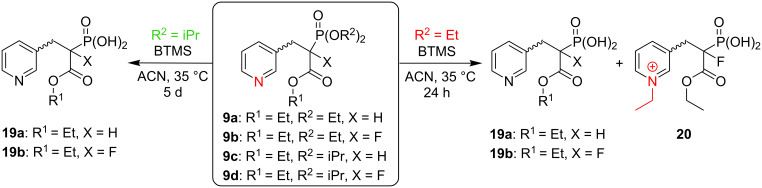
*N*-Alkylation of **9**.

**Scheme 5:**
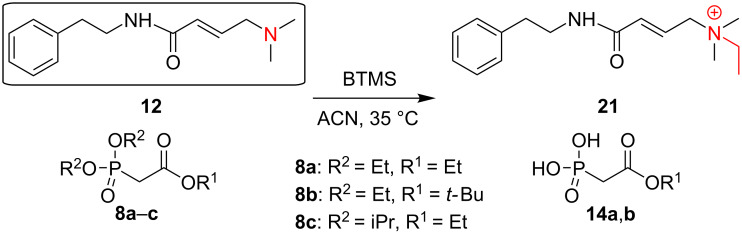
N-Alkylation of **12**.

As reported in the original paper [1], the McKenna reaction required up to 2 h at room temperature for simple phosphonates. However, depending on the structure of the phosphonate, longer reaction times are required, and the reaction is commonly carried out overnight [26–27]. However, during the first step of the McKenna reaction, alkyl bromide is formed (Scheme 1) representing an alkylating agent, which upon prolonged reaction time, may lead to side product formation. In order to study this process, we chose phosphonate analogs **9a**–**d** and acryl amide **12** as the model compounds. While in the case of compounds **9a–d** the source of the alkyl bromide, i.e., a dialkyl phosphonate group already was part of the compound structures, the reactions with compound **12** were performed in the presence of trialkyl phosphonoacetates (Scheme 4 and Scheme 5).

As expected, our studies using phosphonates **9a**,**b** showed that as the concentration of ethyl bromide increases (Scheme 1), the formation of the *N*-alkylated product **20** was observed (Scheme 4), for compound **9a** as well as for the α-fluorinated analog **9b** (7–10% within 24 h, at 35 °C, monitored by ^31^P NMR spectroscopy; see Figure S13 in Supporting Information File 1).

When performing the reaction of **9b** with BTMS under a gentle flow of inert gas (in order to remove the volatile ethyl bromide), we observed a slight decrease in the formation of the *N*-alkylated product **20** (from 7–10% to 5%). However, the flow of the gas needed to be gently controlled to avoid BTMS removal from the reaction mixture.

We encountered a different situation for compound **12**. This substrate underwent *N*-alkylation under the McKenna reaction conditions only in the presence of Et_3_N, a commonly used additive for the protection of unsaturated compounds, such as compound **12** towards HBr addition to the double bond. Within 24 h we observed ≈90% conversion into the aminium salt **21**. Decreasing the amount of Et_3_N, also led to a decrease in the amount of the *N*-alkylated side product (reaching zero when no Et_3_N was added). This indicated that excess Et_3_N makes compound **12** more susceptible to *N*-alkylation. The reason for this observation might be the significantly higher basicity of Et_3_N (p*K*_a_ 10.65) compared to **12**, as approximated based on the p*K*_a_s of representative amines resembling the structure of compound **12**, Me_3_N (p*K*_a_ 9.76) and allyldimethylamine (p*K*_a_ 8.72), respectively [44]. This effect was reinforced by the higher nucleophilicity of the nitrogen atom in **12** compared with Et_3_N, as we deduced by comparison of model tertiary amines: Me_3_N (in MeCN: N 23.05, s_N_ 0.45) and Et_3_N (in MeCN: N 17.10, s_N_ 0.52) [45–46]. On the other hand, the lower nucleophilicity of pyridine compared with the tertiary alkylamine present in compound **12** [45–46] may be responsible for the lower vulnerability of **9** towards alkylation.

Another approach to circumvent the formation of *N*-alkylation products was based on the use of the more sterically hindered diisopropyl phosphonate ester instead of the diethyl analogs. Even though the silylation step required longer times in case of the diisopropyl phosphonate ester [1], which may promote the formation of side products, no *N-*alkylated product was observed, neither for diisopropyl phosphonates **9c**,**d** (even during 13 days), nor for the reaction of compound **12** in the presence of diisopropyl phosphonoacetate **8c**, even with TEA (10 equiv) added (Table 2, entry 4). However, it could not be excluded that this positive outcome would be observed for dimethyl phosphonate esters, due to the low boiling point of methyl bromide (≈4 °C), and therefore its easy removal from the reaction mixture.

**Table 2:** Investigation of the reaction between compound **12**, phosphonoacetates **8** and BTMS.^a^

entry	R^1^	R^2^	TEA	ratio **12**:**21**

1	*t*-Bu	Et	6	0.18:0.82
2	*t*-Bu	Et	3	0.58:0.42
3	*t*-Bu	Et	–	1:0
4	Et	iPr	10	1:0

^a^Reactions carried out for 24 h at 35 °C in the presence of the appropriate phosphonoacetate (see Scheme 5) using distilled BTMS.

### Section 4: Exchange of chloride for bromide

BTMS is known to act as brominating agent for various functional groups, such as alcohols [47–48], three- and four-membered cyclic ethers [49], and anomeric glycosyl acetates [50]. However, to the best of our knowledge, there were no reports on using BTMS for the nucleophilic substitution of alkyl chlorides. A similar exchange reaction was reported only for 2-chloropyridines [51], and required harsh reaction conditions, such as heating at 90–100 °C for 100 h.

**Scheme 6:**
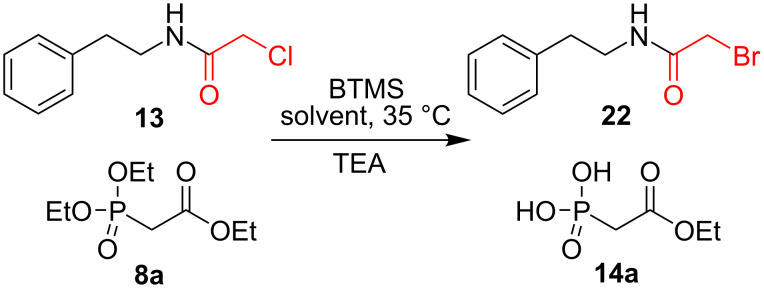
Exchange of the chlorine substituent with bromine in 2-chloro-*N*-phenethylacetamide (**13**) under McKenna reaction conditions.

In this study we investigated the susceptibility of 2-chloro-*N*-phenethylacetamide (**13**), towards halogen exchange mediated by BTMS (Scheme 6). The reaction progress was monitored by ^1^H NMR spectroscopy monitoring the signals of the methylene groups C(O)C*H*_2_Cl in **13** vs C(O)C*H*_2_Br in the product **22** (see Figure S16 in Supporting Information File 1). Further, we confirmed the identity of brominated product **22** by mass spectrometry.

Under these conditions compound **13** underwent an almost complete exchange of chlorine for bromine in the presence of BTMS in MeCN at 35 °C within 3 h (Table 3, entry 1). Changing the solvent to the non-polar aprotic solvent, CDCl_3_ (Table 3, entry 2, 42% conversion after 3 h) as well as lowering the temperature (Table 3, entry 3, rt, CDCl_3_, after 24 h 50% conversion into **22**) slowed down the reaction. When TEA was added to the mixture, the halogen-exchange reaction slightly decreased (Table 3, entries 4 and 5). Remarkably, when performing the reaction without phosphonate added, we isolated only side product **22**, demonstrating that the McKenna reaction and the substitution of chloride were two independent processes (Table 3, entry 6). Based on these results we summarized, that the α-chloroacetamide is susceptible to reaction with BTMS, and underwent exchange of the chlorine substituent for bromine. This reaction can be partially circumvented by using nonpolar solvents, amines and lower temperatures.

**Table 3:** Studies on the reactivity of **13** in the presence of BTMS.^a^

entry	solvent	TEA	PC^b^	time [h]	ratio **13**:**22**

1	ACN	–	+	1 3 24	0.15:0.85 0.02:0.98 0:1
2	CDCl_3_	–	+	3 24	0.58:0.42 0:1
3	CDCl_3_	–	–	24 (rt)	0.5:0.5
4	ACN	1	+	3	0.57:0.43
5	CDCl_3_	1	+	3	0.64:0.36
6	ACN	–	–	24	0:1

^a^The reaction was carried out with distilled BTMS (6 equiv) at 35 °C, except for entry 3, where the reaction was carried out at rt; ^b^PC: trialkyl phosphonocarboxylate **8a**.

### Section 5: Cleavage of the *tert*-butyl carboxyester group

BTMS is known for its chemoselectivity and cleaves phosphonate esters, without affecting carboxyester groups. However, the formation of phosphonic acids in the solvolysis step of the Mc Kenna reaction could lead to the cleavage of acid-labile groups [52–53]. In order to prevent this reaction, the solvolysis step commonly is performed in buffered solutions [53].

In this study, we took a closer look at the reactivity upon exposure to BTMS of two types of carboxyester groups, the acid-labile *tert-*butyl and the relatively stable ethyl esters. For this we chose triethyl phosphonoacetate (**8a**), *tert*-butyl phosphonoacetate (**8b**) and its pyridine-3-ylmethyl analogs **9a**,**e** as the model compounds.

The reaction progress was monitored by ^31^P and ^1^H NMR spectroscopy. The cleavage of the carboxyester group during the first step of the McKenna reaction, was observed as an upfield signal in the ^31^P NMR spectrum (by 0.5–1 ppm), and changes of multiplet signals in the ^1^H NMR spectrum (in case of *tert*-butyl carboxyesters as a decrease in the signal stemming from the *t-*Bu ester group (δ 1.46 ppm) and the appearance of residual signals from isobutylene: δ 1.71 (t) and 4.65 (heptet) ppm; multiplication of signals representing methylene group, bridging carboxylate and phosphonate groups (see Supporting Information File 1, Figures S20–S22). Afterwards, the samples were evaporated and subjected to solvolysis with CD_3_OD/D_2_O (Table 4, last column).

**Table 4:** The stability of compounds **8**/**9** during the McKenna reaction.^a,b^.

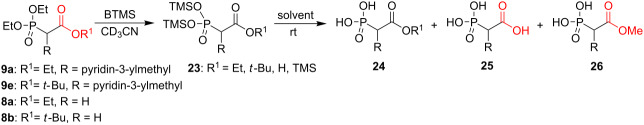							

compound			silylation			solvolysis^c^	

entry	R	R^1^	amine (equiv)	time (h)	ester bond cleavage	BTMS	molar ratio **24**:**25**:**26** (time)

1	H	Et	–	24	0	–	0.98:0.02:0 (24 h)
2	H	Et	–	24	0	+	0.63:0.07:0.30 (18 h)^d^
3	H	*t*-Bu	–	1 24	30% 100%	– –	0:1:0 (1 h) –
4	H	*t*-Bu	TEA (1)	1 24	0 44%	–	– 0.5:0.5:0^e^
5	H	*t*-Bu	pyridine (1 or 2)	24	47%	–	0.5:0.5:0
7	3-Py-CH_2_-	*t*-Bu	–	24	0	–	1:0:0 (24 h)
8	3-Py-CH_2_-	*t*-Bu	–	24	0	+	0:1:0

^a^Reaction carried out in CD_3_CN at rt with 12 equiv BTMS (distilled), unless defined otherwise; ^b^for analogs R = H, the McKenna reaction completed within 1 h; for analogs R = pyridin-3-ylmethyl, the McKenna reaction was completed within 2 h; ^c^the experiment was run on the same sample as the one used for the BTMS studies. For this, the sample was evaporated and directly subjected to solvolysis with MeOD/D_2_O 20:1 (v/v) for 5–10 minutes, unless stated otherwise in parentheses; ^d^solvolysis: MeOH/H_2_O 40:1, BTMS (0.5 equiv); ^e^the same result was obtained after 24 h.

As reported previously [2], when applying BTMS to triethyl phosphonoacetate (**8a**), we did not observe a cleavage of the carboethoxy group during the silylation step (Table 4, entry 1). However, the result of the subsequent solvolysis of the thus formed trimethylsilyl ester **23**, depended on the conditions applied. The exposure of **23** to acetone or methanol, gave only the expected product **24** (up to 24 h). However, when a mixture of methanol and water (20:1, v/v) was used, traces (2%) of the fully hydrolyzed product **25** were detected, but only after 24 h (Table 4, entry 1), while the solvolysis usually takes a few minutes.

Trying to simulate the conditions of a not appropriately carried out McKenna reaction, i.e., BTMS not thoroughly evaporated before solvolysis, we added BTMS at the solvolysis stage to the mixture of methanol and water (40:1, v/v). In this case we observed a partial ethyl ester cleavage (up to 7%), and its transesterification into methyl ester **26** (up to 30% within 18 h, Table 4, entry 2).

Next we turned to the vulnerability of the *tert*-butyl ester group to BTMS. While iodotrimethylsilane is known for its capability to cleaving carboxyester groups [12,54], with *tert*-butyl and benzyl esters being more susceptible than methyl or ethyl esters, no such reaction was reported in the presence of BTMS. To our surprise we observed a partial cleavage of the *tert*-butyl ester group present in phosphonoacetate **8b** already at the silylation step (30% within 1 h; 100% after 24 h; Table 4, entry 3). In order to exclude the possibility that this reaction was caused by the presence of trace amounts of HBr, we tested Et_3_N (1 equiv) or pyridine (1 or 2 equiv) as scavengers. Although the reaction slowed down, still a significant cleavage of the *tert*-butyl ester bond was detected within 24 h (Table 4, entries 4 and 5). This observation might imply that this reaction was similar to the one between carboxyesters and ITMS, and proceeded via a silylated ester bromide salt intermediate [12,54].

In a further experiment we subjected *tert*-butyl ester **9e** to BTMS action. This model compound contained a pyridine ring embedded in the structure and in this case no cleavage of the *tert*-butyl group was observed (Table 4, entry 7). This result was opposite to the one obtained for *tert-*butyl phosphonoacetate **8b** in the presence of pyridine (Table 4, entry 5). At this point the role of pyridine, either added to the mixture or embedded within the carboxyester molecules, remained unclear.

## Conclusion

The McKenna reaction is one of the most robust methods for the mild deprotection of organophosphorus esters. However, the benefits of this reaction are sometimes overshadowed by side reactions taking place when polyfunctional organophosphorus compounds are involved. Here, we evaluated experiments that did not work, the so called “dark data” [21], and we demonstrated that contrary to common belief: 1) BTMS itself led to the cleavage of *tert*-butyl carboxyesters, and 2) amines, a commonly used standard additives in the McKenna reaction were not generally safe and may promoted side reactions in the presence of certain functional groups. Besides that, we discovered two new applications of BTMS, providing new mild methods for the synthesis of oxazoles and an α-bromoacetamide. Even though the former synthesis was not completely chemoselective, the pure oxazoles could be easily isolated in good yields, and the proposed procedure constitutes an interesting alternative to existing methods, which usually require harsh conditions.

## Experimental

Knowing the sensitivity of the McKenna reaction to water and oxygen, all equipment and reagents were dried prior to use and the reactions were run under an inert argon atmosphere, unless specified otherwise. CH_3_CN/CD_3_CN and CHCl_3_/CDCl_3_ were dried using activated 3 Å and 4 Å molecular sieves. In all experiments, unless specified otherwise, we used commercial BTMS, which was distilled and placed in ampules under Ar, and stored frozen in the dark until use. Triethylamine and pyridine were distilled and stored over sodium hydroxide, All reagents, including synthesized and commercially available phosphonoacetates were dried with appropriate drying agents and under high vacuum. In most cases, the reaction progress, of both, the silylation step using BTMS as well as the solvolysis step, were monitored by ^1^H and ^31^P NMR spectroscopy, using deuterated solvents, CD_3_CN, CDCl_3_, CD_3_OD, or D_2_O. The progress of the McKenna reaction can easily be monitored by ^31^P NMR spectroscopy, where the exchange of each alkyl for trimethylsilyl group in the phosphonate involves a ≈8–10 ppm upfield shift of signals in the spectrum. The side products were isolated and their structures confirmed by NMR spectroscopy and HRMS. Mass spectra were recorded in the positive ion mode with an Agilent 6220 mass spectrometer coupled with an Agilent 1200 series HPLC. NMR spectra were measured at 250.13 or 700 MHz for ^1^H NMR, 62.90 or 170 MHz for ^13^C NMR, and 283 or 101.30 MHz for ^31^P NMR on a Bruker Avance DPX 250 or a Bruker Avance II Plus 700 spectrometer, respectively.

### General procedures for the McKenna reaction (recommended)

All reactions were run under an Ar atmosphere, using dry equipment, dried solvents and reagents. In a dried round-bottomed flask equipped with a septum, the appropriate substrate was placed (1 equiv) and dissolved in ACN or CHCl_3_ (50 mg/1.5 mL). Then, TEA (1 equiv, as specified in the text; the addition of TEA was required only in certain cases) was added, followed by BTMS (6–8 equiv). The septum was exchanged by a fitted glass stopper and additionally secured with parafilm. The flask was placed in a sand bath at 36 °C for 24 h. Afterwards, the solution was evaporated and the mixture subjected to solvolysis, using either acetone, ethanol or methanol/water (20:1, v/v).

The isolation of phosphonic acids as the main products of the McKenna reaction was not the topic of this investigation, and most model substrates that were used in the study did not contain organophosphorus ester/acid. If required (compounds **20** and **21**), the product was purified by HPLC. Additionally, the free phosphonic acid could be easily removed from the reaction mixture by washing with aqueous sodium carbonate solution.

## Supporting Information

File 1 Synthesis of starting materials, copies of ^31^P NMR, ^1^H NMR, and ^13^C NMR spectra for all new compounds and selected NMR spectra illustrating the formation of the side products.
