# Supplementary material for: The McKenna reaction – avoiding side reactions in phosphonate deprotection
Source: Beilstein J Org Chem. 2020 Jun 23;16:1436–46. doi: 10.3762/bjoc.16.119 (PMC7323628; doi:10.3762/bjoc.16.119)
Supplement: File 1 — Synthesis of starting materials, copies of 31P NMR, 1H NMR, and 13C NMR spectra for all new compounds and selected NMR spectra illustrating the formation of the side products. [file Beilstein_J_Org_Chem-16-1436-s001.pdf]

BEILSTEIN JOURNAL OF ORGANIC CHEMISTRY

Supporting Information

for

The McKenna reaction – avoiding side reactions in phosphonate deprotection

Katarzyna Justyna, Joanna Małolepsza, Damian Kusy, Waldemar Maniukiewicz and Katarzyna M. Błażewska

Beilstein J. Org. Chem. **2020,** *16,* 1436–1446. doi:10.3762/bjoc.16.119

Synthesis of starting materials, copies of ^{31}P NMR, ^{1}H NMR, and ^{13}C NMR spectra for all new compounds and selected NMR spectra illustrating the formation of the side products

License and Terms: This is a supporting information file under the terms of the Creative Commons Attribution License (http://creativecommons.org/licenses/by/4.0). Please note that the reuse, redistribution and reproduction in particular requires that the authors and source are credited.
The license is subject to the *Beilstein Journal of Organic Chemistry* terms and conditions: (https://www.beilstein-journals.org/bjoc)

Table of contents

1. **Synthesis of substrates** S3

1.1. **4-Nitro-*N*-(prop-2-yn-1-yl)benzamide (10)** S3

1.2. ***N*-Phenethylacrylamide (11)**: S3

1.3. **General procedure: Synthesis of phosphonocarboxylate analogues of risedronate** (**9a-e**) S3

1.4. **2-Chloro-*N*-phenethylacetamide (13)** S6

1.5. **4-(Dimethylamino)-*N*-phenethylbut-2-enamide (12)** S6

2. **Synthesis of side products under McKenna reaction conditions** S7

2.1. Synthesis of compounds **15–17** S7

2.2. **3-Bromo-*N*-phenethylpropanamide** (**18**) S8

2.3. **3-(3-Ethoxy-2-fluoro-3-oxo-2-phosphonopropyl)-1-ethylpyridin-1-ium** (**20**) S8

2.4. **2-Bromo-*N*-phenethylacetamide** (**22**) S9

2.5. ***N*-Ethyl-*N*,*N*-dimethyl-4-oxo-4-(phenethylamino)but-2-en-1-aminium** (**21**) S9

3. **NMR Spectra of side products generated under Mc Kenna reaction conditions** S11

3.1. **Compounds 15–17** S11

Figure S1: ^{1}H NMR of compound **15** ($CDCl_3$, 700 MHz). S11

Figure S2: ^{13}C NMR of compound **15** ($CDCl_3$, 176 MHz). S12

3.1.1. X-ray Crystallography of compound **15** S12

Figure S3: Molecular structure of **15** from X-ray crystallographic analysis. S13

Table S1. Crystal and structure refinement data. S13

Figure S4: ^{1}H NMR of compound **16** and **17** ($CDCl_3$, 700 MHz). S14

Figure S5: ^{1}H NMR of compound **16** and **17** ($CDCl_3$, 700 MHz): zoom in of the range 4.2–8.2. ..... S15

Figure S6: ^{13}C NMR of compound **16** and **17** ($CDCl_3$, 176 MHz). S15

3.2. Optimization of oxazole synthesis S16

Table S2: Optimization of the oxazole synthesis.[a] S16

3.3 Compound 18 ..... S17

Figure S7: ^{1}H NMR of compound **18** ($CDCl_3$, 700 MHz). S17

Figure S8: ^{13}C NMR of compound **18** ($CDCl_3$, 176 MHz). S17

3.4 Compound 20 ..... S18

Figure S9: ^{1}H NMR of compound **20** ($CDCl_3$, 700 MHz). S18

Figure S10: ^{1}H NMR of compound **20** ($CDCl_3$, 700 MHz): zoom in of the ranges 1.1–1.7, 3.6–4.8, and 7.9–8.9 ppm. S18

Figure S11: ^{13}C NMR of compound **20** ($CDCl_3$, 176 MHz). S19

Figure S12: ^{31}P NMR of compound **20** ($CDCl_3$, 283 MHz)........ S19

Figure S13: Top and middle: Monitoring of the alkylation reaction progress for compound **9b** at the first stage of the McKenna reaction (BTMS stage); top: ^{31}P NMR after 24 h; middle: ^{31}P NMR after 7 days. Bottom: ^{31}P NMR spectrum of the alkylation product **20** after solvolysis and HPLC purification. S20

3.5 Compound 22........ S21

Figure S14: ^{1}H NMR of compound **22** ($CDCl_3$, 700 MHz). S21

Figure S15: ^{13}C NMR of compound **22** ($CDCl_3$, 176 MHz). S21

Figure S16: Monitoring by ^{1}H NMR of the reaction progress between **13** and BTMS (CD_3CN, 700 MHz): zoom in of the ranges 2.7–4.3 ppm........ S22

3.6 Compound 21........ S23

Figure S17: ^{1}H NMR of compound **21** (CD_3OD, 700 MHz). S23

Figure S18: ^{13}C NMR of compound **21** (CD_3OD, 176 MHz). S24

Figure S19: Comparison of the ^{1}H NMR spectra of substrate **12** (bottom spectrum) and product **21** (top spectrum; compound contaminated with ≈ 10% of substrate **12**) (CD_3OD + TFA, 700 MHz): zoom in of the ranges 1.25–4.20, and 6.30–7.35 ppm. The representative peaks were identified. . S24

3.7 Monitoring the experiments for section 5 by NMR........ S25

Figure S20: Monitoring of the reaction between **8b** and BTMS: top: ^{1}H NMR spectrum of **8b** before adding BTMS; middle: ^{1}H NMR after 24 h of reaction between **8b** and BTMS (CD_3CN, 700 MHz); bottom: ^{1}H NMR after 5 min of solvolysis (CD_3OD, 700 MHz); zoom in of the ranges 3.45–2.7 and 2.0–1.2 ppm; see Table 4, entry 3........ S25

Figure S21: Monitoring of the reaction between **8b** and BTMS in the presence of 1 equiv of TEA: top: ^{1}H NMR spectrum of **8b** before adding BTMS; middle: ^{1}H NMR after 24 h of reaction between **8b** and BTMS (CD_3CN, 700 MHz); bottom: ^{1}H NMR after 5 min of solvolysis (CD_3OD, 700 MHz); zoom in of the ranges 3.15–2.7 and 2.0–1.25 ppm; see Table 4, entry 4........ S25

Figure S21a: Monitoring the reaction between **8b** and BTMS in the presence of 1 equiv of TEA: top: ^{31}P NMR spectrum of **8b** before adding BTMS; middle: ^{31}P NMR after 24 h of reaction between **8b** and BTMS (CD_3CN, 700 MHz); bottom: ^{31}P NMR after 5 min of solvolysis (CD_3OD, 700 MHz); zoom in of the ranges 3.15–2.7 and 2.0–1.25 ppm; see Table 4, entry 4........ S26

Figure S22: Monitoring the reaction between **8b** and BTMS in the presence of 2 equiv of pyridine: top: ^{1}H NMR spectrum of **8b** before adding of BTMS; middle: ^{1}H NMR after 24 h of reaction between **8b** and BTMS (CD_3CN, 700 MHz); bottom: ^{1}H NMR after 5 min of solvolysis (CD_3OD, 700 MHz); zoom in of the ranges 3.5–2.7 and 2.0–1.25 ppm; see Table 4, entry 5........ S26

4. References........ S27

1. Synthesis of substrates

1.1. 4-Nitro-*N*-(prop-2-yn-1-yl)benzamide (10)[1]

The reaction was run under an inert gas atmosphere (Ar). In a dried round-bottomed flask equipped with a septum propargylamine hydrochloride (740 mg, 8.08 mmol, 1.5 equiv) and triethylamine (12.4 mL) in 22 mL of dry dichloromethane were placed. Then the mixture was cooled to −40 °C and a solution of 4-nitrobenzoyl chloride (1 g, 5.39 mmol, 1 equiv) in dry CH_2Cl_2 (22 mL) was added dropwise. The reaction was stirred for 24 h at room temperature. Afterwards the reaction was quenched by the addition of water (7.4 mL). After 10 minutes, additional 22 mL of water were added and the mixture extracted with $CHCl_3$ (3 × 80 mL). The combined extracts were dried ($MgSO_4$) and concentrated to give the crude product, which was purified by column chromatography (DCM/acetone 0–30%). Yield 1.080 g (98%) from 1 g (5.39 mmol) of 4-nitrobenzoyl chloride.

^{1}H NMR ($CDCl_3$, 700 MHz): δ_H 2.32 (1H, t, *J* = 2.6 Hz, CH_2CCH), 4.28 (2H, dd, *J* = 5.2, 2.6 Hz, CH_2CCH), 6.33 (1H, bs, CO-NH), 7.91-7.98 (2H, m, CH_{Ar}), 8,26-8,34 (m, 2H, CH_{Ar}).

1.2. *N*-Phenethylacrylamide (11):[2]

The reaction was run under inert gas atmosphere (Ar). In a dried round-bottomed flask equipped with a septum 2-phenethylamine (1 g, 8.25 mmol, 1 equiv) in 15 mL of dry dichloromethane was placed. Then the mixture was cooled to −50 °C and triethylamine (24.8 mmol, 3 equiv) and acryloyl chloride (1.494 g, 16.5 mmol, 2 equiv) were added. The reaction was stirred for 1.5 h at −50 °C. Then water was added and the mixture extracted with $CHCl_3$ (3 × 20 mL) at pH 9 (adjusted by Na_2CO_3 (sat.), if needed). The combined extracts were dried over ($MgSO_4$) and concentrated to give the crude product, which was purified by flash column chromatography (hexane/AcOEt 0–50%). Yield 743 mg (51.4%) from 1 g (8.25 mmol) of 2-phenethylamine.

^{1}H NMR ($CDCl_3$, 250 MHz): δ_H 2.86 (2H, t, *J* = 6.9 Hz, Ar-CH_2-CH_2), 3.61 (2H, td, *J* = 7.0, 6.0 Hz, Ar-CH_2-CH_2), 5.56-5.66 (1H, m, CH=CH_2), 5.95-6.10 (1H, m, CH=CH_2), 6.21-6.31 (1H, m, CH=CH_2), 7.15-7.37 (5H, m, CH_{Ar}).

1.3. General procedure:[3] Synthesis of phosphonocarboxylate analogues of risedronate (9a-e)

Step I: The reaction was run under inert gas atmosphere (Ar). In a dried round-bottomed flask equipped with a septum the appropriate phosphonoacetate (1 equiv) in 15 mL of dry

dichloromethane (0.3 M solution) was placed. The mixture was cooled to −40 °C and $TiCl_4$ (1 equiv) and triethylamine (2.8 equiv) were added slowly, maintaining the temperature constant. After 15 minutes, 3-pyridinealdehyde was added dropwise. The reaction mixture was stirred for 30 min at −40 °C, followed by 24 h at room temperature. Then, 30 mL of water and saturated Na_2CO_3 were added to adjust pH to around 9 and the mixture was extracted with $CHCl_3$ (5 × 30 mL). The combined extracts were dried ($MgSO_4$) and concentrated to give the crude product, which was purified by flash column chromatography (DCM/acetone 0–30%).

Step II: In a round-bottomed flask the appropriate vinyl analogue of phosphonocarboxylate (1 equiv) in MeOH (0.1 M solution) was placed. Then, the $NiCl_2 \cdot 6H_2O$ (1.2 equiv) was added, the mixture cooled to −40 °C and $NaBH_4$ added in portions until the substrate disappeared as monitored by mass spectrometry. The reaction was quenched by the addition of a saturated aqueous solution of NH_4Cl and the mixture extracted with CH_2Cl_2 (4 × 60 mL) at pH 9 (adjusted with Na_2CO_3(sat.)). The combined extracts were dried ($MgSO_4$) and concentrated to give the crude product, which was purified by flash column chromatography (DCM/acetone 0–50%).

Step III: The reaction was run under inert gas atmosphere (Ar). In a dried round-bottomed flask equipped with a septum NaH (1.2 equiv; 60% dispersion in mineral oil) in 10 mL of dry THF (0.32 M solution) was placed. The mixture was cooled to −15 °C and a solution of the appropriate phosphonopropionate (1 equiv) in 7 mL of dry THF (0.23 M solution) was added dropwise and the resulting mixture was stirred for 50 min at 0 °C. Next, the reaction mixture was cooled to −78 °C and a solution of NFSI (1.5 equiv) in dry THF (0.34 M solution) added dropwise. The reaction mixture was stirred for 20 min at −60 °C, followed by 1.5 h at −20 to (−40) °C. At −20 °C, 5 mL of a saturated aqueous solution of NH_4Cl and 5 mL of water were added and when the mixture had reached rt it was extracted with $CHCl_3$ (4 × 20 mL). The combined extracts were dried ($MgSO_4$) and concentrated to give the crude product, which was purified by flash column chromatography (DCM/acetone).

Ethyl 2-(diethoxyphosphoryl)-3-(pyridin-3-yl)propanoate (9a):[4] Yield 726 mg (72 %) from 1 g (3.19 mmol) of ethyl 2-(diethoxyphosphoryl)-3-(pyridin-3-yl)acrylate; **^{1}H NMR** ($CDCl_3$, 250 MHz): δ_H 1.15 (3H, t, J = 7.1 Hz, COOCH$_2$C$\underline{H_3}$), 1.34 (6H, t, J = 7.1 Hz, PO(OCH$_2$C$\underline{H_3}$)$_2$), 3.11-3.34 (3H, m, Py-C$\underline{H_2}$-C$\underline{H}$), 4.00-4.30 (6H, m, COOC$\underline{H_2}$CH$_3$ and PO(OC$\underline{H_2}$CH$_3$)$_2$), 7.20 (1H, ddd, J = 7.8, 4.8, 0.9 Hz, Py), 7.53 (1H, dt, J = 7.8, 2.0 Hz, Py), 8.43-8.49 (2H, m, Py). **^{31}P NMR** ($CDCl_3$, 101 MHz): δ_P 21.43 (s).[52]

Ethyl 2-(diethoxyphosphoryl)-2-fluoro-3-(pyridin-3yl)propanoate (9b): Yield 378 mg (71 %) from 500 mg (1.59 mmol) of ethyl 2-(diethoxyphosphoryl)-3-(pyridin-3-yl)propanoate; **^{1}H NMR** ($CDCl_3$, 700 MHz):[52] δ_H 1.19 (3H, t, *J* = 7.1 Hz, $COOCH_2C\underline{H_3}$), 1.37 (6H, q, *J* = 7.0 Hz, $PO(OCH_2C\underline{H_3})_2$), 3.41-3.56 (2H, m, Py-C$\underline{H_2}$-CF), 4.16-4.32 (6H, m, $COOC\underline{H_2}CH_3$ and $PO(OC\underline{H_2}CH_3)_2$), 7.22 (1H, ddd, *J* = 7.8, 4.8, 0.9 Hz, Py), 7.58-7.52 (1H, m, Py), 8.47-8.48 (1H, m, Py), 8.51 (1H, dd, *J* = 4.8, 1.7 Hz, Py). **^{31}P NMR** ($CDCl_3$, 283 MHz): δ_P 11,88 (d, J_{PF} = 82 Hz).

Ethyl 2-(diisopropoxyphosphoryl)-3-(pyridin-3-yl)propanoate (9c): Yield 2.106 g (76 %) from 2.737 g (8.02 mmol) of ethyl 2-(diisopropoxyphosphoryl)-3-(pyridin-3-yl)acrylate; **^{1}H NMR** ($CDCl_3$, 700 MHz): δ_H 1.13 (3H, t, *J* = 7.1 Hz, $COOCH_2C\underline{H_3}$), 1.32 (12H, dt, *J* = 9.6, 5.9 Hz, $PO(OCH(C\underline{H_3})_2)_2$), 3.09-3.24 (3H, m, Py-C$\underline{H_2}$-C$\underline{H}$), 4.03-4.11 (2H, m, $COOC\underline{H_2}CH_3$), 4.69-4.76 (2H, m, $PO(OC\underline{H}(CH_3)_2)_2$), 7.15-7.18 (1H, m, Py), 7.48-7.51 (1H, m, Py), 8.43 (1H, dd, *J* = 4.8, 1.7 Hz, Py), 8.44 (1H, d, *J* = 2,4 Hz, Py). **^{13}C NMR** ($CDCl_3$, 176 MHz): δ_C 14.10, 23.85 (d, *J* = 5.2 Hz), 23.97 (d, *J* = 5.1 Hz), 24.08 (d, *J* = 4.0 Hz), 24.24 (d, *J* = 3.3 Hz), 30.36 (d, *J* = 4.1 Hz), 48.19 (d, *J* = 131 Hz), 61.51, 71.68 (d, *J* = 7.1 Hz), 71.93 (d, *J* = 6.9 Hz), 123.40, 134.37 (d, *J* = 15.9 Hz), 136.25, 148.25, 150.22, 168.30 (d, *J* = 5.1 Hz). **^{31}P NMR** ($CDCl_3$, 283 MHz): δ_P 18.92 (s).

Ethyl 2-(diisopropoxyphosphoryl)-2-fluoro-3-(pyridin-3-yl)propanoate (9d): Yield 200 mg (38 %) from 500 mg (1.46 mmol) of ethyl 2-(diisopropoxyphosphoryl)-3-(pyridin-3-yl)propanoate; **^{1}H NMR** ($CDCl_3$, 700 MHz): δ_H 1.17 (3H, t, *J* = 7.1 Hz, $COOCH_2C\underline{H_3}$), 1.31-1.38 (12H, m, $PO(OCH(C\underline{H_3})_2)_2$), 3.37-3.51 (2H, m, Py-C$\underline{H_2}$-CF), 4.12-4.20 (2H, m, $COOC\underline{H_2}CH_3$), 4.76-4.88 (2H, m, $PO(OC\underline{H}(CH_3)_2)_2$), 7.19 (1H, ddd, *J* = 7.8, 4.8, 0.9 Hz, Py), 7.55-7.59 (1H, m, Py), 8.44 (1H, d, *J* = 2.0 Hz, Py), 8.48 (1H, dd, *J* = 4.8, 1.7 Hz, Py). **^{13}C NMR** ($CDCl_3$, 176 MHz): δ_C 14.08, 23.71 (d, *J* = 5.6 Hz), 23.81 (d, *J* = 5.3 Hz), 24.23 (d, *J* = 3.3 Hz), 24.34 (d, *J* = 2.8 Hz), 36.72 (d, *J* = 19.7 Hz), 62.46, 73.57 (d, *J* = 7.1 Hz), 73.81 (d, *J* = 7.0 Hz), 123.31, 129.36 (d, *J* = 12.7 Hz), 137.93, 148.94, 151.26, 166.38 (d, *J* = 3.9 Hz), 166.51 (d, *J* = 3.9 Hz). **^{31}P NMR** ($CDCl_3$, 283 MHz): δ_P 10.01 (d, J_{PF} = 84.5 Hz).

***tert*-Butyl 2-(diethoxyphosphoryl)-3-(pyridin-3-yl)propanoate (9e):** Yield 1.049 g (35 %) from 3 g (8.80 mmol) of tert-butyl 2-(diethoxyphosphoryl)-3-(pyridin-3-yl)acrylate; **^{1}H NMR** ($CDCl_3$, 700 MHz): δ_H 1.30 (9H, s, $COOC(C\underline{H_3})_3$), 1.32 (6H, t, *J* = 7.1 Hz, $PO(OCH_2C\underline{H_3})_2$), 3.07-3.22 (3H, m, Py-C$\underline{H_2}$-C$\underline{H}$), 4.11-4.19 (4H, m, $PO(OC\underline{H_2}CH_3)_2$), 7.15-7.18 (1H, m, Py), 7.50-7.53 (1H, m, Py), 8.42-8.44 (1H, m, Py), 8.44-8.46 (1H, m, Py). **^{13}C NMR** ($CDCl_3$, 176

MHz): δ_C 16.49 (m, 2C), 27.86 (3C), 30.22 (d, *J* = 4.0 Hz), 48.16 (d, *J* = 129.6 Hz), 62.86 (d, *J* = 7.0 Hz), 62.95 (d, *J* = 6.4 Hz), 82.32, 123.33, 134.31 (d, *J* = 16.5 Hz), 136.31, 148.23, 150.27, 167.16 (d, *J* = 5.0 Hz). **^{31}P NMR** ($CDCl_3$, 283 MHz): δ_P 21.72 (s).

1.4. 2-Chloro-*N*-phenethylacetamide (13)

(The procedure is based on a modified method from literature.[5] NMR signals correspond with literature data). The reaction was run under inert gas atmosphere (Ar). In a dried round-bottomed flask equipped with a septum 2-phenethylamine (200 mg, 1.65 mmol, 1 equiv) in 3 mL of dry dichloromethane was placed. Then, the mixture was cooled to −50 °C, and triethylamine (9.90 mmol, 3 equiv) and chloroacetyl chloride (373 mg, 3.30 mmol, 2 equiv) were added and the resulting reaction mixture was stirred for 1.5 h at −50 °C. Then, water was added and the mixture extracted with $CHCl_3$ (3 × 20 mL) at pH 9. The combined extracts were dried ($MgSO_4$) and concentrated to give the crude product. After washing the chloroform solution 3 times with water (acidified with 15% HCl; pH ≈ 4–5) the pure product was obtained. Yield 315 mg (96%) from 200 mg (1.65 mmol) of 2-phenethylamine.

^{1}H-NMR ($CDCl_3$, 250 MHz): δ_H 2.85 (1H, t, *J* = 7.0 Hz, Ar-C$\underline{H_2}$-CH_2), 3.57 (2H, td, *J* = 7.0, 5.9 Hz, Ar-CH_2-C$\underline{H_2}$), 4.03 (2H, s, C$\underline{H_2}$-Cl), 6.60 (1H, bs, N$\underline{H}$-CO), 7.15-7.40 (5H, m, CH_{Ar}).

1.5. 4-(Dimethylamino)-*N*-phenethylbut-2-enamide (12)

The reaction was run under inert gas atmosphere (Ar). In a dried round-bottomed flask equipped with a septum 4-(dimethylamino)-2-butenoic acid hydrochloride (205 mg, 1.24 mmol, 1.5 equiv) in 1.5 mL of dry DMF was placed. Then, the mixture was cooled to −20 °C and HATU (1.24 mmol, 1.5 equiv) and DIPEA (1.24 mmol, 1.5 equiv) were added and the mixture stirred for 5 min. Then, 2-phenethylamine (100 mg, 0.82 mmol, 1 equiv) and DIPEA (1.24 mmol, 1.5 equiv) were added and the mixture warmed to rt and stirred overnight. The reaction was quenched by the addition of water and extracted with $CHCl_3$ (3 × 5 mL). The combined extracts were dried ($MgSO_4$) and concentrated to give the crude product, which was purified by flash column chromatography ($CHCl_3$/MeOH 0–10 %). Yield 139 mg (73%) from 100 mg (0.82 mmol) of 2-phenethylamine.

^{1}H NMR (CD_3OD, 700 MHz): δ_H 2.68 (6H, s, N-(C$\underline{H_3}$)$_2$), 2.83 (2H, t, *J* = 7.4 Hz, Ar-C$\underline{H_2}$-CH_2), 3.49 (2H, t, *J* = 7.4 Hz, Ar-CH_2-C$\underline{H_2}$), 3.64 (2H, dd, *J* = 7.1, 1.4 Hz, C$\underline{H_2}$-N-(CH_3)$_2$), 6.23 (1H, dt, *J* = 15.3, 1.4 Hz, CO-CH=C$\underline{H}$), 6.70 (1H, dt, *J* = 15.3, 7.1 Hz, CO-C$\underline{H}$=CH), 7.17-7.20 (1H, m, $CH_{Ar(4)}$), 7.20-7.23 (2H, m, $CH_{Ar(3,5)}$), 7.25-7.29 (2H, m, $CH_{Ar(2,6)}$). **^{13}C NMR**

(CD_3OD, 176 MHz): δ_C 36.36 (Ar-$\underline{C}H_2$-CH_2), 42.14 (Ar-CH_2-$\underline{C}H_2$), 43.93 (2C, N-($\underline{C}H_3)_2$), 59.58 ($\underline{C}H_2$-N-($CH_3)_2$), 127.40 ($C_{Ar(4)}$), 129.50 ($C_{Ar(2,6)}$), 129.76 ($C_{Ar(3,5)}$), 131.78 (CO-CH=$\underline{C}$H), 134.14 (CO-$\underline{C}$H=CH), 140.35($C_{Ar(1)}$), 166.79 (C=O). **LRMS** (APCI) m/z: [M + H]+ Calcd for $C_{14}H_{20}N_2O$ 232.16; Found 232.9.

2. Synthesis of side products under McKenna reaction conditions

2.1. Synthesis of compounds **15–17**

The reactions were run under inert gas atmosphere (Ar). In a dried round-bottomed flask equipped with a septum 4-nitro-*N*-(prop-2-yn-1-yl)benzamide (**10**, 0.245 mmol, 1 equiv) was placed and dissolved in dry ACN (50 mg/1.5 mL). Then, triethyl phosphonoacetate (0.245 mmol, 1 equiv) and H_2O (0.490 mmol, 2 equiv) were subsequently added, followed by BTMS (2.94 mmol, 12 equiv). The septum was exchanged with a fitted glass stopper and additionally secured with parafilm. After 24 h in a 36 °C sand bath, the solution was evaporated and the mixture was subjected to solvolysis in acetone. After 5 min, the solvent was evaporated, the residue dissolved in $CHCl_3$ and extracted with $NaHCO_3$ (2 × 2,5 mL). The combined extracts were dried ($MgSO_4$) and concentrated to give the crude product, which was purified by flash column chromatography (CH_2Cl_2).

5-Methyl-2-(4-nitrophenyl)oxazole (**15**):[6] **^{1}H NMR** ($CDCl_3$, 700 MHz): δ_H 2.43 (3H, d, *J* = 1.2 Hz, $CH_{Ar(5)}$-$C\underline{H_3}$), 6.93 (1H, q, *J* = 1.2 Hz, $CH_{Ar(4)}$), 8.12-8.16 (2H, m, $CH_{Ar(2',6')}$), 8.27-8.30 (2H, m, $CH_{Ar(3',5')}$). **^{13}C NMR** ($CDCl_3$, 176 MHz): δ_C 11.25 ($C_{Ar(5)}$-$\underline{C}H_3$), 124.27 ($C_{Ar(3',5')}$), 125.50 ($C_{Ar(4)}$), 126.68 ($C_{Ar(2',6')}$), 133.26 ($C_{Ar(4')}$), 148.45 ($C_{Ar(1')}$), 150.85 ($C_{Ar(5)}$), 158.74 ($C_{Ar(2)}$). **HRMS** (DART+) m/z: [M + H]+ Calcd for $C_{10}H_9N_2O_3$ 205.0608; Found 205.0609.

***N*-(2-Bromoallyl)-4-nitrobenzamide** and ***N*-(3-bromoallyl)-4-nitrobenzamide** (**16** and **17**): **^{1}H NMR** ($CDCl_3$, 700 MHz): δ_H 4.19 (2H, td, *J* = 6.0, 1.5 Hz, CO-NH-$C\underline{H_2}$ (**17**)), 4.28 (2H, d, *J* = 6.0 Hz CO-NH-$C\underline{H_2}$ (**16**)), 5.56 (1H, d, *J* = 2.2 Hz, CBr=$C\underline{H_2}$), 5.84-5.86 (1H, m, CBr=$C\underline{H_2}$), 6.29 (1H, q, *J* = 6.5 Hz, CH=C$\underline{H}$Br), 6.33 (1H, dt, *J* = 7.1, 1.5 Hz, C$\underline{H}$=CHBr), 7.12 (1H, t, *J* = 5.8 Hz, CO-N$\underline{H}$ (**17**)), 7.23 (1H, d, *J* = 6.2 Hz, CO-N$\underline{H}$ (**16**)), 7.92-7.99 (2H, m, $CH_{Ar(2,6)}$), 8.19-8.25 (2H, m, $CH_{Ar(3,5)}$). **^{13}C NMR** ($CDCl_3$, 176 MHz): δ_C 39.89 (CO-NH-$\underline{C}H_2$ (**17**)), 48.10 (CO-NH-$\underline{C}H_2$ (**16**)), 110.95 (CH=$\underline{C}$HBr), 118.66 (CBr=$\underline{C}H_2$), 123.83, 123.88 (2s, $C_{Ar(3)}$ and $C_{Ar(5)}$), 128.38, 128.45 (2s, $C_{Ar(2)}$ and $C_{Ar(6)}$), 128.82 ($\underline{C}$Br=CH_2), 130.53 ($\underline{C}$H=CHBr), 139.63, 139.76 (2s, $C_{Ar(1)}$ or $C_{Ar(4)}$), 149.66, 149.76 (2s, $C_{Ar(1)}$ or $C_{Ar(4)}$), 165.63 (s, $\underline{C}$O (**17**)),

165.85 (s, CO (**16**)). **HRMS** (DART+) m/z: [M + H]+ Calcd for $C_{10}H_{10}N_2O_3Br$ 284.9869; Found 284.9874.

2.2. 3-Bromo-*N*-phenethylpropanamide (18)

The reaction was run under inert gas atmosphere (Ar). In a dried round-bottomed flask equipped with a septum *N*-phenethylacrylamide (**11**, 0.285 mmol, 1 equiv) was placed and dissolved in dry ACN (50 mg/1.5 mL). Then, triethyl phosphonoacetate (0.285 mmol, 1 equiv) and H_2O (0.143 mmol, 0.5 equiv) were subsequently added, followed by BTMS (not distilled, 3.42 mmol, 12 equiv). The septum was exchanged with a fitted glass stopper and additionally secured with parafilm. After 24 h in a 36 °C sand bath the solution was evaporated and the mixture was subjected to solvolysis in acetone. After 5 min, the solvent was evaporated, the residue dissolved in $CHCl_3$ and extracted with $NaHCO_3$ (2 × 2.5 mL). The extract was dried ($MgSO_4$) and concentrated to give the crude product, which was purified by flash column chromatography (DCM/acetone 0–10%).

^{1}H NMR ($CDCl_3$, 700 MHz):[7] δ_H 2.69 (2H, t, *J* = 6.6 Hz, CO-CH_2-CH_2Br), 2.83 (2H, t, *J* = 7.0 Hz, Ph-CH_2-CH_2), 3.54 (2H, td, *J* = 7.0, 5.9 Hz, Ph-CH_2-CH_2), 3.61 (2H, t, *J* = 6.6 Hz, CO-CH_2-CH_2Br), 5.75 (1H, br s, NH-CO), 7.20 (2H, br d, *J* = 6.6 Hz, $CH_{Ar(2,6)}$), 7.23, (1H, br t, *J* = 7.4 Hz, $CH_{Ar(4)}$), 7.31 (2H, br *t*, *J* = 7.6 Hz, $CH_{Ar(3,5)}$). **^{13}C NMR** ($CDCl_3$, 176 MHz): δ_C 27.58 (CO-CH_2-CH_2Br), 35.71 (Ph-CH_2-CH_2), 39.85 (CO-CH_2-CH_2Br), 40.89 (Ph-CH_2-CH_2), 126.68 ($C_{Ar(4)}$), 128.77 ($C_{Ar(3,5)}$), 128.88 ($C_{Ar(2,6)}$), 138.80 ($C_{Ar(1)}$), 169.71 (CO). **HRMS** (DART+) m/z: [M + H]+ Calcd for $C_{11}H_{15}NOBr$ 256.0332; Found 256.0328.

2.3. 3-(3-Ethoxy-2-fluoro-3-oxo-2-phosphonopropyl)-1-ethylpyridin-1-ium (20)

The reaction was run under inert gas atmosphere (Ar). In a dried round-bottomed flask equipped with a septum ethyl 2-(diethoxyphosphoryl)-2-fluoro-3-(pyridin-3-yl)propanoate (**9d**, 0.51 mmol, 1 equiv) was placed and dissolved in dry ACN (170 mg/5 mL). Then, BTMS (3.06 mmol, 6 equiv) was added and the septum was exchanged with a fitted glass stopper and additionally secured with parafilm. After 13 days in a 36 °C sand bath the solution was evaporated and the mixture was subjected to solvolysis in EtOH. After 5 min, the solvent was evaporated to give the crude product, which was purified by HPLC (95% H_2O + 5% ACN + 0.2% TFA buffer, isocratic).

^{1}H NMR (D_2O, 700 MHz, pH ≈ 2-3): δ_H 1.21 (3H, t, *J* = 7.1 Hz, O-CH_2-CH_3), 1.62 (3H, t, *J* = 7.4 Hz, N-CH_2-CH_3), 3.64-3.80 (2H, m, CH_2-CF), 4.20-4.28 (2H, m, O-CH_2-CH_3), 4.59-4.67

(2H, m, N-C$\underline{H_2}$-CH_3), 8.01 (dd, 1H, *J* = 8.1, 6.16 Hz, $CH_{Ar(5)}$), 8.43 (d, 1H, *J* = 8.2 Hz, $CH_{Ar(4)}$), 8.79 (dt, 1H, *J* = 6.2, 1.3 Hz, $CH_{Ar(2)}$), 8.83 (d, 1H, *J* = 1.9 Hz, $CH_{Ar(6)}$). **^{13}C NMR** (D_2O, 176 MHz, pH ≈ 2-3): δ_C 13.33 (O-CH_2-$\underline{C}H_3$), 15.70 (N-CH_2-$\underline{C}H_3$), 36.05 (d, J_{FC} = 19.8 Hz, $\underline{C}H_2$-CF), 57.44 (N-$\underline{C}H_2$-CH_3), 63.66 (O-$\underline{C}H_2$-CH_3), 95.87 (dd, J_{FC} = 194.6 Hz, J_{PC} = 145.5 Hz, CH_2-$\underline{C}$F), 127.94 ($C_{Ar(5)}$), 136.19 (d, J_{FC} = 11.7 Hz, $C_{Ar(3)}$), 142.95 ($C_{Ar(6)}$), 144.52 ($C_{Ar(2)}$), 146.8 ($C_{Ar(4)}$), 169.63 (dd, J_{FC} = 23.3 Hz, J_{PC} = 2.7 Hz, -$\underline{C}$OOEt). **^{31}P NMR** (D_2O, 283 MHz, pH ≈ 2-3) δ_P 6.31 (d, J_{PF} = 71,3 Hz). **LRMS** (ESI) m/z: [M]+ Calcd for $C_{12}H_{18}FNO_5P$ 306.09; Found 305.9.

2.4. 2-Bromo-*N*-phenethylacetamide (22)

The reaction was run under inert gas atmosphere (Ar). In a dried round-bottomed flask equipped with a septum 2-chloro-*N*-phenethylacetamide (**13**, 0.051 mmol, 1 equiv) was placed and dissolved in dry $CDCl_3$ (10.1 mg/0.5 mL). Then, BTMS (0.306 mmol, 6 equiv) was added and the septum was exchanged with a fitted glass stopper and additionally secured with parafilm. After 24 h in a 36 °C sand bath the solution was evaporated and the mixture was subjected to solvolysis in acetone. After 5 min the solvent was evaporated.

^{1}H NMR ($CDCl_3$, 700 MHz):[8] δ_H 2.90 (2H, t, *J* = 7.1 Hz, Ph-C$\underline{H_2}$-CH_2), 3.58 (2H, br m, Ph-CH_2-C$\underline{H_2}$), 4.03 (2H, s, C$\underline{H_2}$Br), 7.18-7.25 (3H, m, $CH_{Ar(3,5)}$ and $CH_{Ar(4)}$), 7.28-7.33 (2H, m, $CH_{Ar(2,6)}$). **^{13}C NMR** ($CDCl_3$, 176 MHz): δ_C 27.48 ($\underline{C}H_2$Br), 35.03 (Ph-$\underline{C}H_2$-CH_2), 42.32 (Ph-CH_2-$\underline{C}H_2$), 126.95 ($C_{Ar(4)}$), 128.87 and 128.89 (2s, $C_{Ar(2,6)}$ and $C_{Ar(3,5)}$), 137.92 ($C_{Ar(1)}$), 168.12 ($\underline{C}$O). **HRMS** (DART+) m/z: [M + H]+ Calcd for $C_{10}H_{13}NOBr$ 242.0175; Found 242.0181.

2.5. *N*-Ethyl-*N,N*-dimethyl-4-oxo-4-(phenethylamino)but-2-en-1-aminium (21)

The reaction was run under inert gas atmosphere (Ar). In a dried round-bottomed flask equipped with a septum 4-(dimethylamino)-*N*-phenethylbut-2-enamide (**12**, crude product, without purification, 0.258 mmol, 1 equiv) was placed and dissolved in dry ACN (60 mg/2 mL). Then, *tert*-butyl diethylphosphonoacetate (0.258 mmol, 1 equiv) and TEA (20.02 mmol, 10 equiv) were subsequently added, followed by BTMS (not distilled, 20.02 mmol, 10 equiv). The septum was exchanged with a fitted glass stopper and additionally secured with parafilm. After 24 h in a 36 °C sand bath the solution was evaporated and the mixture was subjected to solvolysis in EtOH. After 5 min the solvent was evaporated and the mixture purified by HPLC (A: 95% H_2O + 5% ACN + 0.2% TFA; B: 95% ACN + 5% H_2O + 0.2% TFA, gradient 5→30 min 0→90% B, compound **21** was collected after 15 min.

^{1}H NMR (CD_3OD, 700 MHz): δ_H 1.39 (3H, t, *J* = 7.3 Hz, N-CH_2-$C\underline{H_3}$), 2.85 (2H, t, *J* = 7.3 Hz, Ar-$C\underline{H_2}$-CH_2), 3.07 (6H, s, N-($C\underline{H_3}$)$_2$), 3.41 (2H, q, *J* = 7.3 Hz, N-$C\underline{H_2}$-CH_3), 3.52 (2H, t, *J* = 7.4 Hz, Ar-CH_2-$C\underline{H_2}$),4.09 (2H, d, *J* = 6.4 Hz, $C\underline{H_2}$-N-(CH_3)$_2$), 6.39-6.43 (1H, m, CO-CH=C$\underline{H}$), 6.77 (1H, dt, *J* = 15.1, 7.5 Hz, CO-C$\underline{H}$=CH), 7.17-7.24 (3H, m, $CH_{Ar(4)}$ and $CH_{Ar(3,5)}$), 7.25-7.30 (2H, m, $CH_{Ar(2,6)}$). **^{13}C NMR** (CD_3OD, 176 MHz): δ_C 8.37 (N-CH_2-$\underline{C}H_3$), 36.30 (Ar-$\underline{C}H_2$-CH_2), 42.40 (Ar-CH_2-$\underline{C}H_2$), 50.55 (2C, t, *J* = 3.8 Hz, N-($\underline{C}H_3$)$_2$), 61.21 (N-$\underline{C}H_2$-CH_3), 64.79 ($\underline{C}H_2$-N-(CH_3)$_2$), 127.45 ($C_{Ar(4)}$), 129.01 (CO-$\underline{C}$H=CH), 129.52 ($C_{Ar(2,6)}$), 129.76 ($C_{Ar(3,5)}$), 135.93 (CO-CH=$\underline{C}$H), 140.29 ($C_{Ar(1)}$), 166.13 (C=O). **HRMS** (DART+) m/z: [M + H]+ Calcd for $C_{16}H_{25}N_2O$ 261.1961; Found 261.1962.

3. NMR Spectra of side products generated under Mc Kenna reaction conditions

3.1. Compounds 15–17

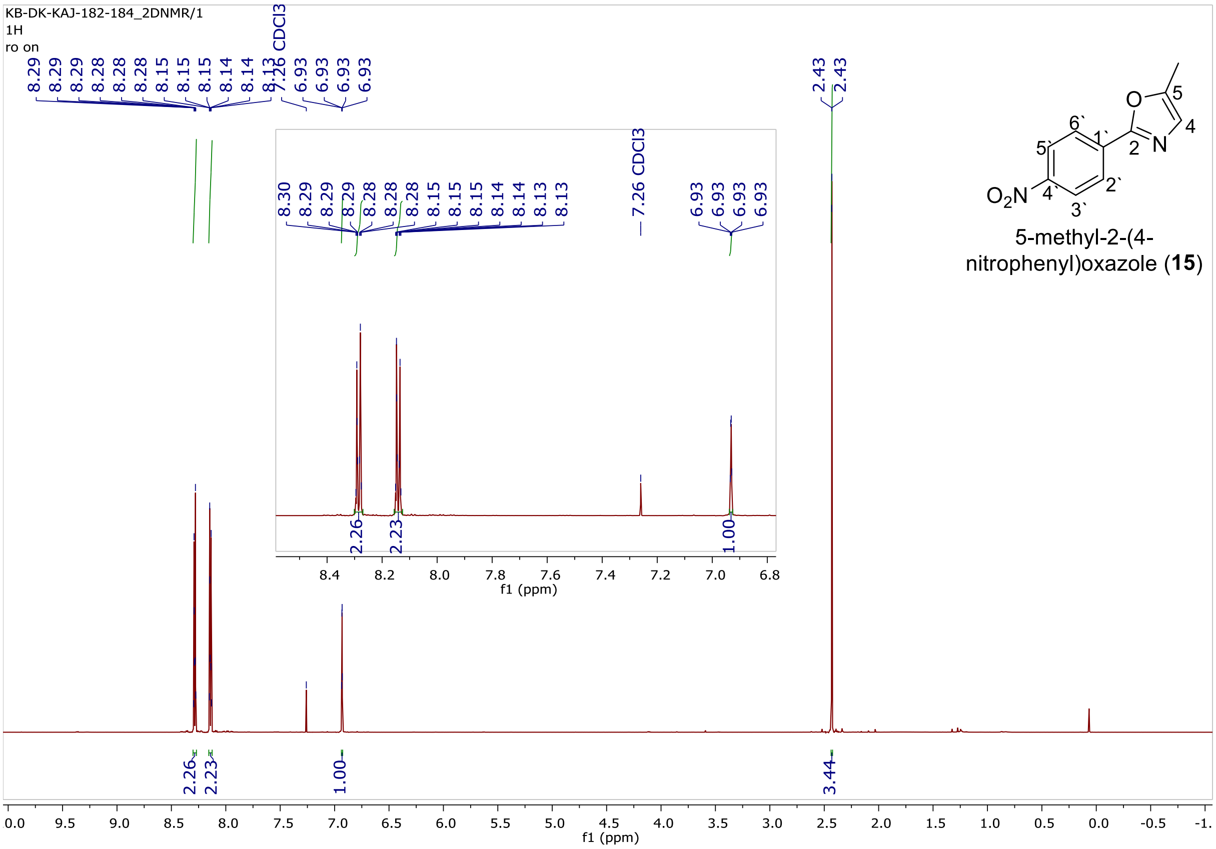

Figure S1: ^{1}H NMR of compound **15** ($CDCl_3$, 700 MHz).

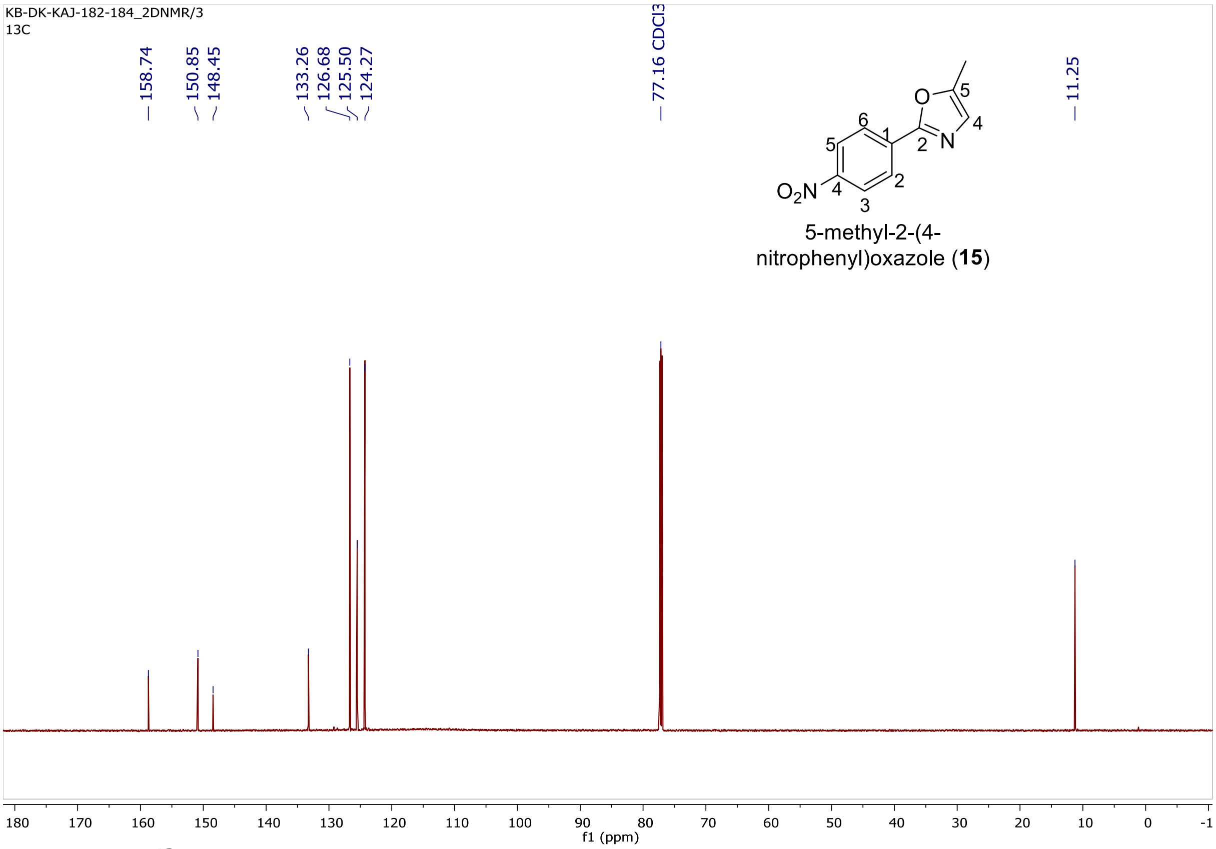

Figure S2: ^{13}C NMR of compound **15** ($CDCl_3$, 176 MHz).

3.1.1. X-ray Crystallography of compound **15**

X-ray data of single crystals of the title compound were measured on an APEX II CCD X-ray diffractometer using graphite-monochromated CuKα radiation (λ = 1.54184 Å). Data were collected using the APEX-II software[9], integrated using SAINT[10] and corrected for absorption using the multi-scan approach (SADABS).[11] Final cell constants were determined from full least squares refinements of all observed reflections. The structures were solved using intrinsic phasing (SHELXT)[12] and refined with full squares refinement on F^2 using the SHELXTL software.[13,14] All hydrogen atoms were added at calculated positions and refined isotropically with a riding model. A thermal ellipsoid plot of the title compound is given in Figure S3. A summary of the experimental crystallographic data is presented in Table S1. The crystallographic data (CCDC 1919198) have been deposited in the Cambridge Crystallographic Data Base.

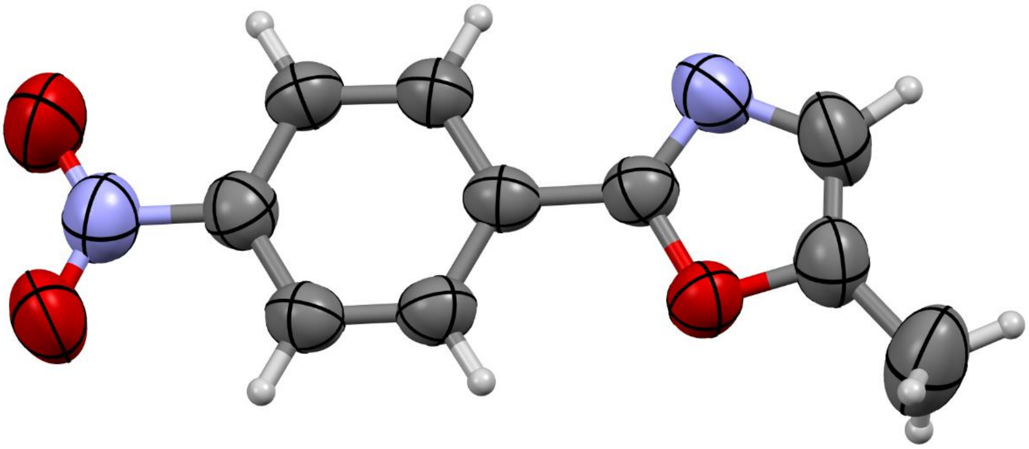

Figure S3: Molecular structure of **15** from X-ray crystallographic analysis. (Thermal ellipsoids are shown at the 50% probability level.)

Table S1. Crystal and structure refinement data.

Formula	$C_{10}H_8N_2O_3$
CCDC no.	1919198
Formula weigh	204.18
Temperature, [K]	298
Wavelength, [Å]	CuK$_\alpha$
Crystal system	triclinic
Space group	*P*1
Unit cell dimensions, [Å, °]	a= 3.9699(2)
	b= 5.9126(2)
	c=10.7094(7)
	α= 98.052(4)
	β= 99.989(5)
	γ= 91.441(4)
Volume, [Å^3]	244.81(2)
Z	1
Calculated density, [g cm^3]	1.385
Absorption coefficient [mm^{-1}]	0.883
θ range for data collection [°]	4.2 - 66.6
Limiting indices (h, k, l)	-4/4, -7/7, -12/12
Reflections collected/unique	3570/1560
Data/restraints/parameters	1560/0/138
R_{int}	0.013
Goodness-of-fit on F^2	1.27
$R[F^2 > 2(F^2)]$	0.0751
R (all data)	0.0888
$wR[F^2 > 2(F^2)]$	0.254
Largest diff. peak and hole, [e Å^{-3}]	-0.37, 0.49

Note

Since compounds **16** and **17** were isolated as mixture, for the description of NMR data symbols (**16** and **17**) were used whenever distinction was possible. When two complementary signals overlapped, no distinction was made.

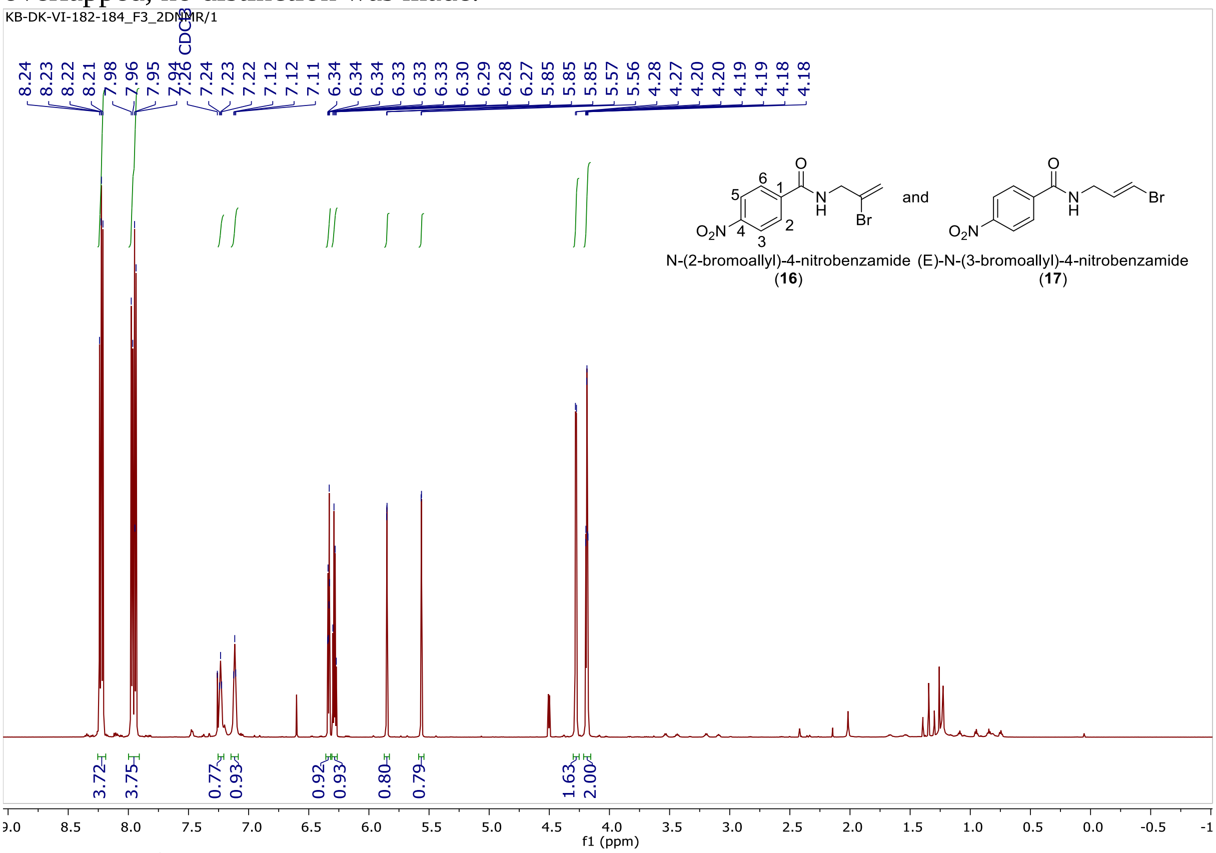

Figure S4: ^{1}H NMR of compound **16** and **17** ($CDCl_3$, 700 MHz).

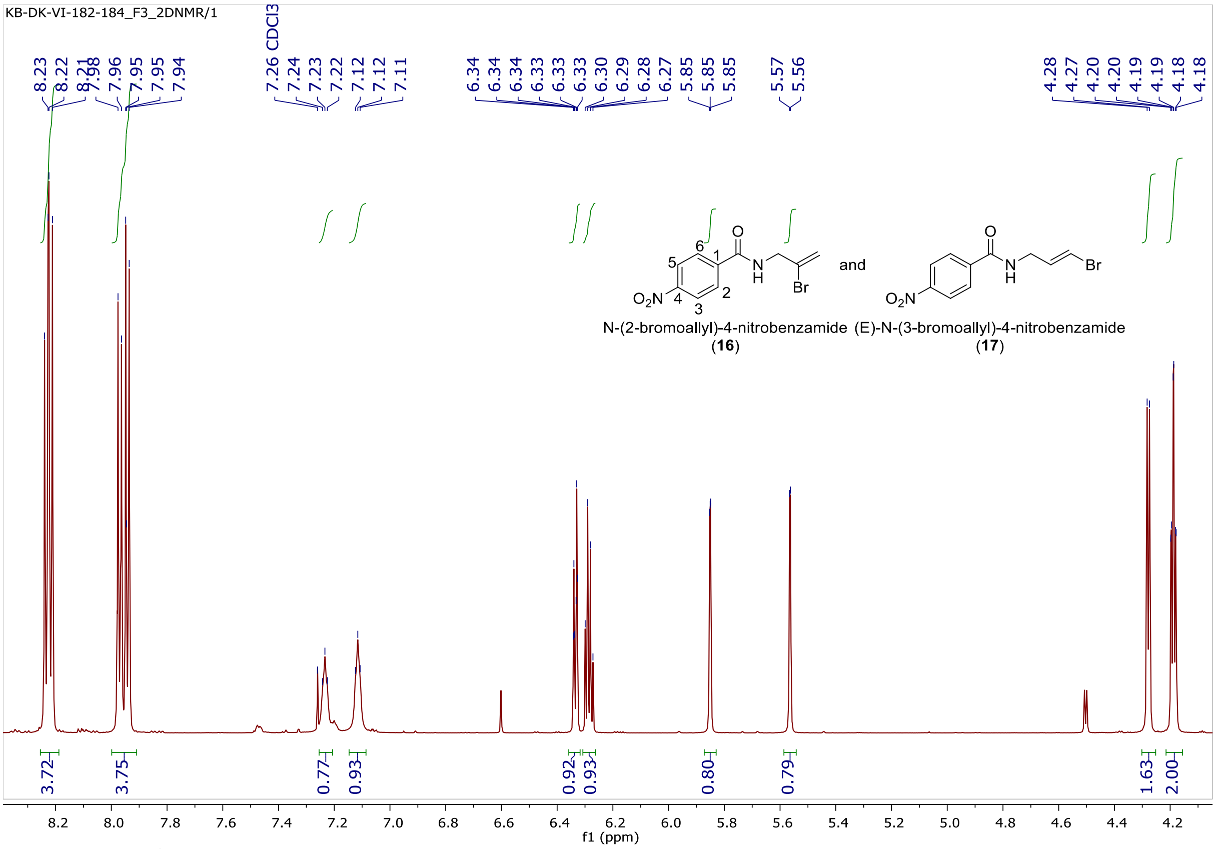

Figure S5: ^{1}H NMR of compound **16** and **17** ($CDCl_3$, 700 MHz): zoom in of the range 4.2–8.2.

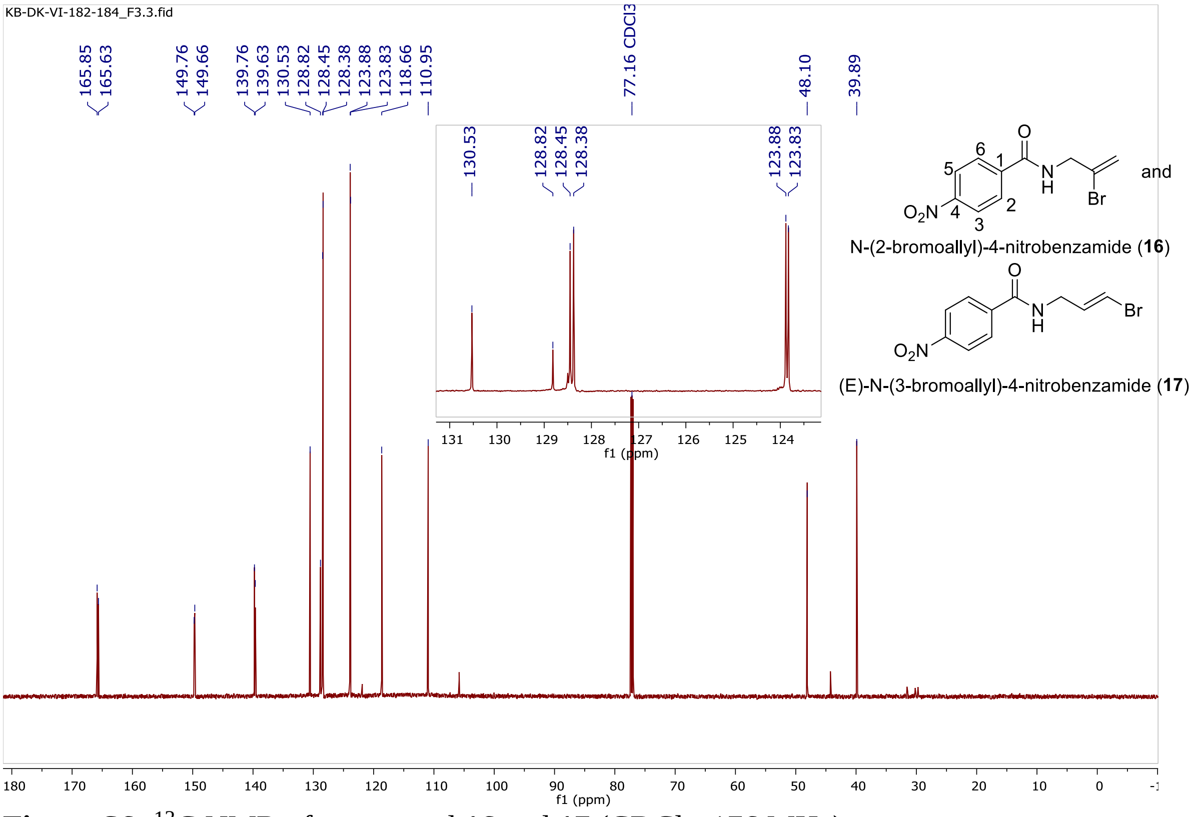

Figure S6: ^{13}C NMR of compound **16** and **17** ($CDCl_3$, 176 MHz).

3.2. Optimization of oxazole synthesis

In the first approach we generated HBr by the reaction between equimolar amounts of BTMS and water (0.5:0.5 and 3:3) (Table S2, entry 2 and 3). We obtained mixture of products and substrate, achieving higher conversion for the 3:3 molar ratio of reagents.

Next, we excluded BTMS from the reaction and used 33% HBr in AcOH or 40% HBr in H_2O, respectively. The application of the HBr solution in AcOH led again to a mixture of products **15–17** and the substrate (Table S2, entries 4–6), while using solely hydrobromic acid (40%) led to recovery of the substrate (Table S2, entry 7).

To exclude the formation of products by HBr addition, we applied non-HBr containing acids: HCl, TFA, and AcOH. However, in all cases we isolated only the starting compound **10** (Table S2, entries 8–11).

Table S2: Optimization of the oxazole synthesis.[a]

Entry	PC[b]	Acid (eq)	Additive (eq)	**10:15:16:17**
**1.**	+	BTMS (0.5)	-	0.96:0.04:0:0
**2.**	+	BTMS (0.5)	H_2O (0.5)	0.7:0.27:0.01:0.02
**3.**	-	BTMS (3)	H_2O (3)	0.13:0.64:0.13:0.10
**4.**	+	33 % HBr in AcOH (1)	-	0.67:0.27:0.03:0.03
**5.**	+	33 % HBr in AcOH (10)	-	0,18:0,51:0,12:0,19
**6.**	+	33 % HBr in AcOH (1)	H_2O (1)	0.34:0.54:0.08:0.04
**7.**	+	40 % HBr in H_2O (10)	-	0,99:0,01:0:0
**8.**[c]	-	AcOH (1)	D_2O (2)	n.r.
**9.**	-	TFA (1.28)	-	n.r.
**10.**	-	TFA (1.28)	H_2O (1.28)	n.r.
**11**	-	HCl (sat) (5 µl)	-	n.r.

[a]All reactions were carried out in ACN; the reaction was run at 35 °C for 24 h. n.r.: no reaction; [b]triethyl phosphonoacetate was used; [c]ACN-d_3 was used, because of the NMR control of this reaction.

3.3 Compound 18[15]

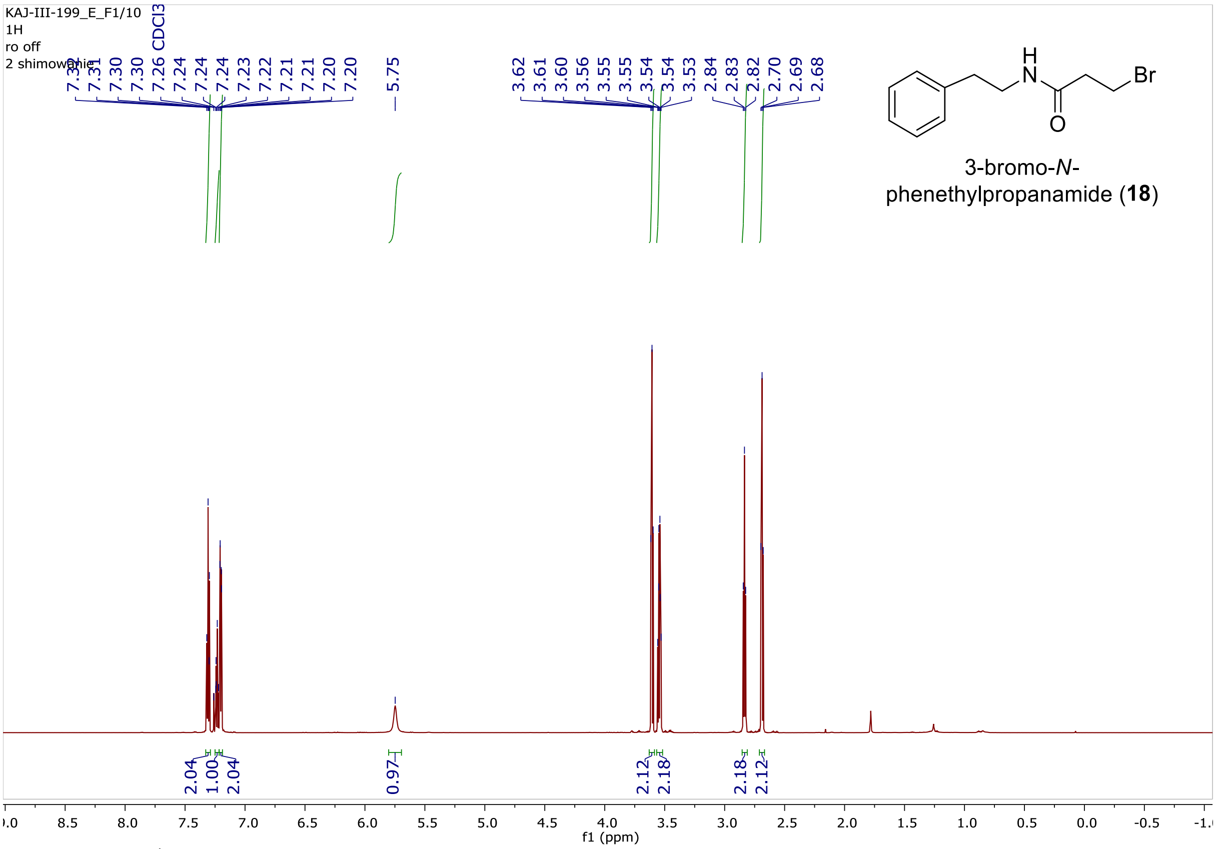

Figure S7: ^{1}H NMR of compound **18** ($CDCl_3$, 700 MHz).

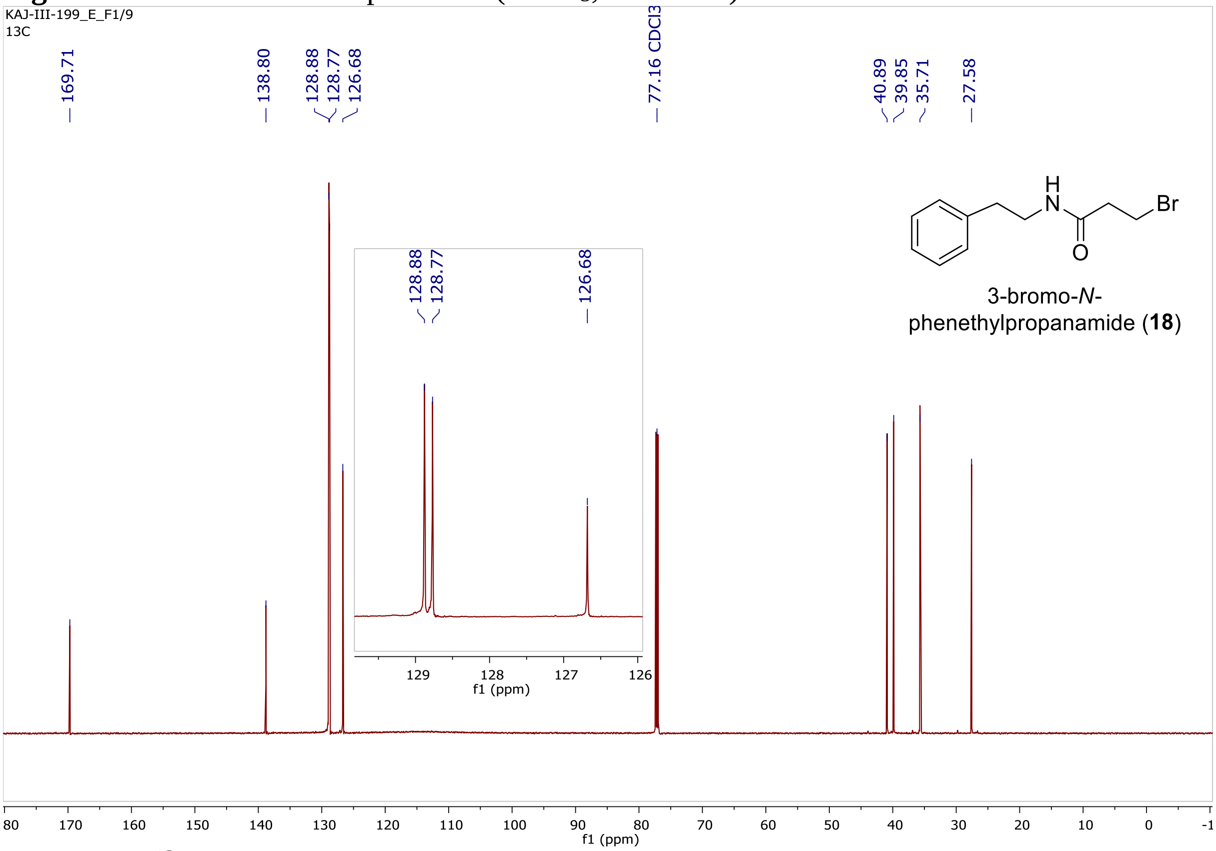

Figure S8: ^{13}C NMR of compound **18** ($CDCl_3$, 176 MHz).

3.4 Compound 20

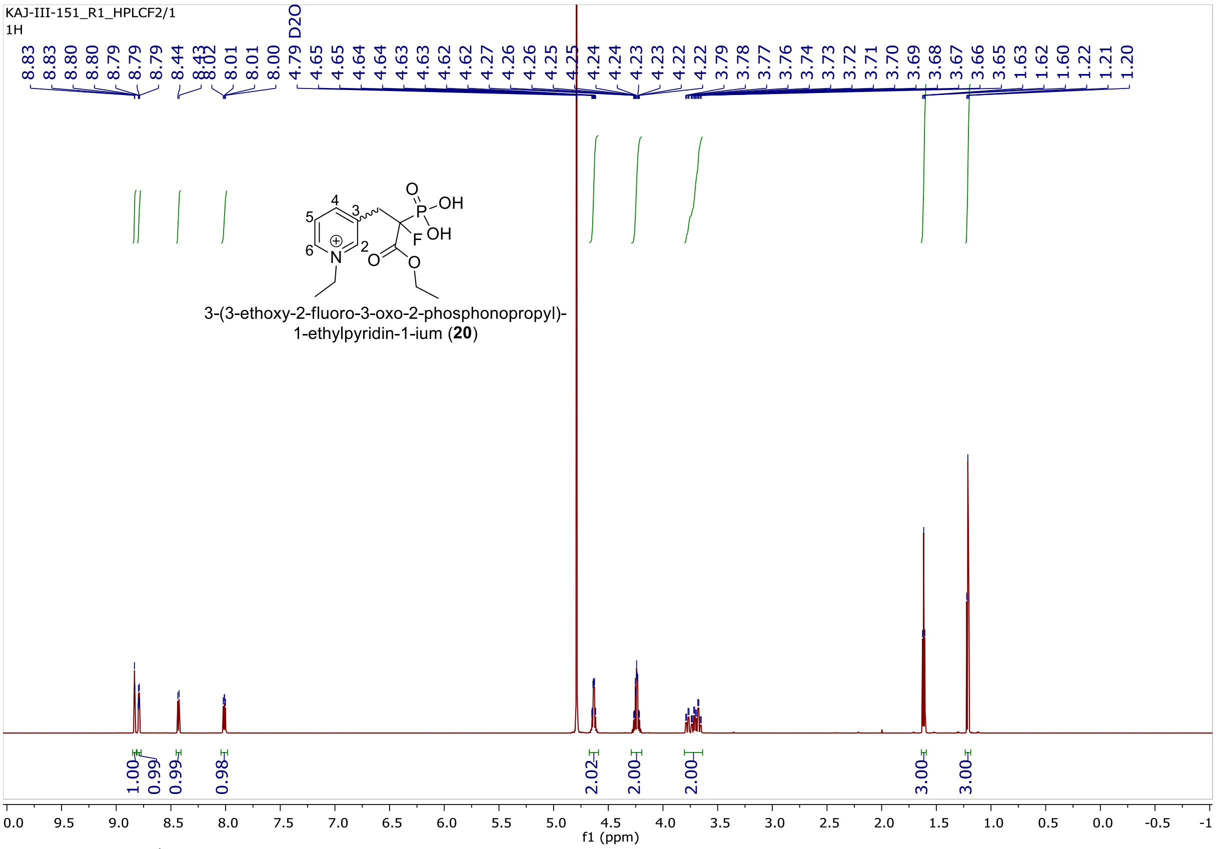

Figure S9: ^{1}H NMR of compound **20** ($CDCl_3$, 700 MHz).

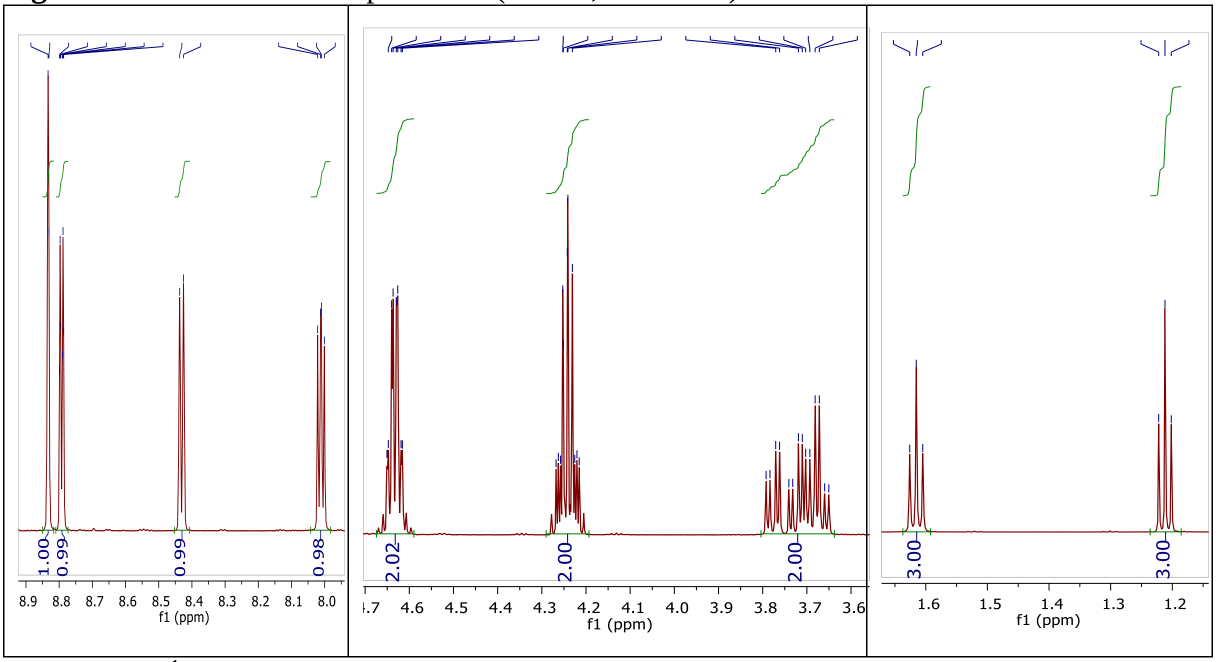

Figure S10: ^{1}H NMR of compound **20** ($CDCl_3$, 700 MHz): zoom in of the ranges 1.1–1.7, 3.6–4.8, and 7.9–8.9 ppm.

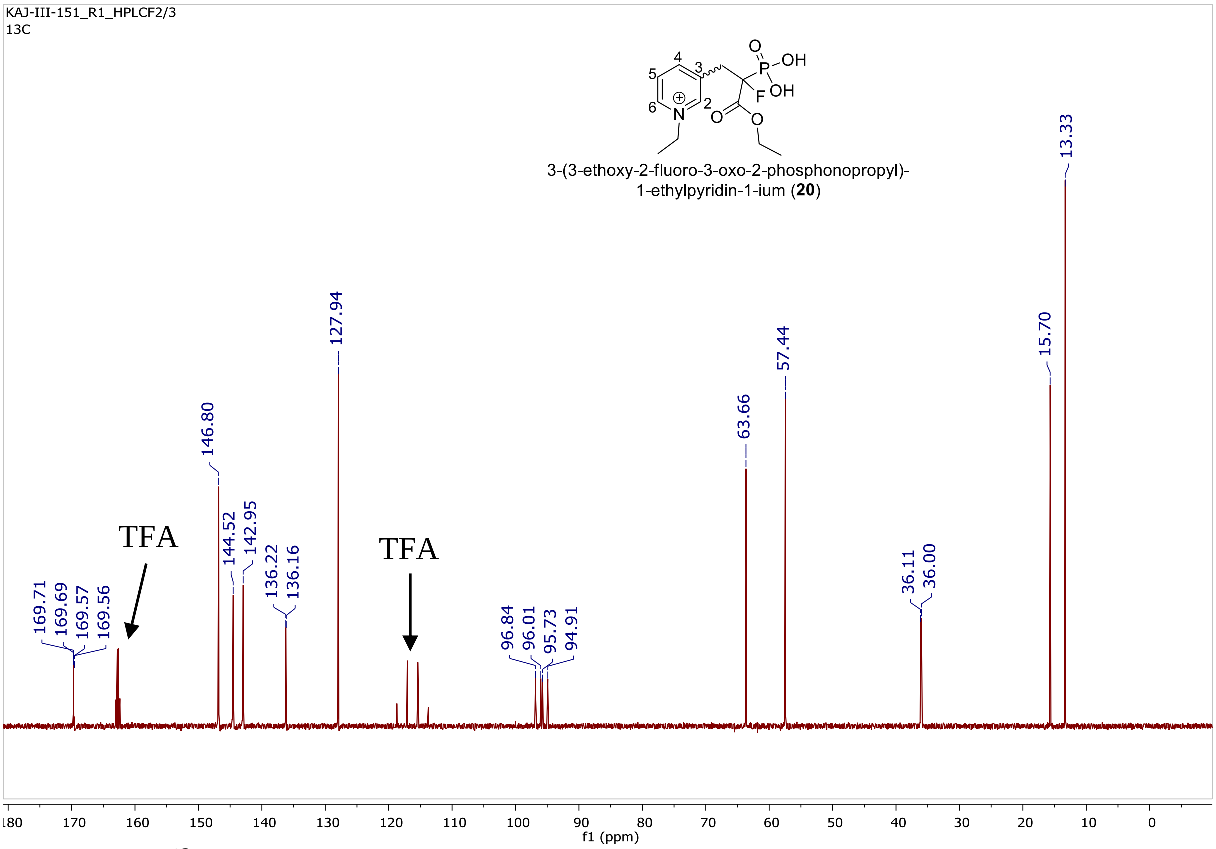

Figure S11: ^{13}C NMR of compound **20** ($CDCl_3$, 176 MHz).

Note: Compound **20** was purified by HPLC using 95% H_2O + 5% ACN + 0.2% TFA buffer. The signals of residual TFA were identified in the ^{13}C NMR spectrum.

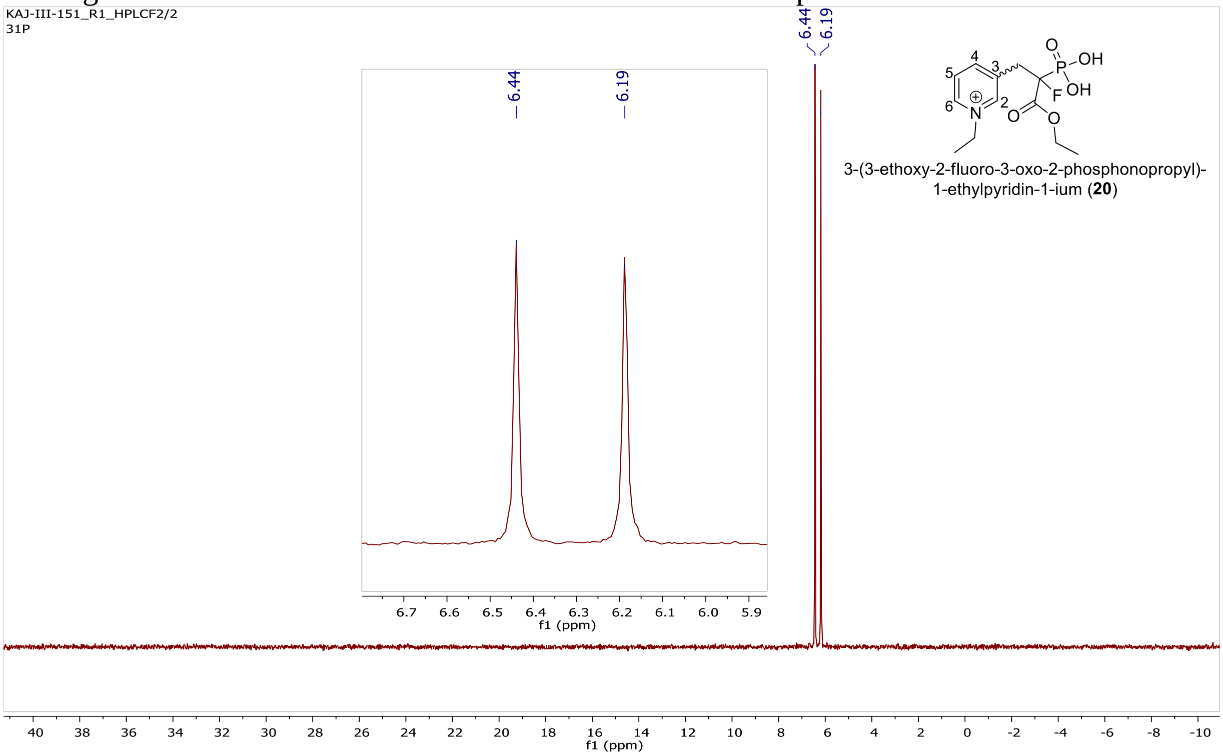

Figure S12: ^{31}P NMR of compound **20** ($CDCl_3$, 283 MHz).

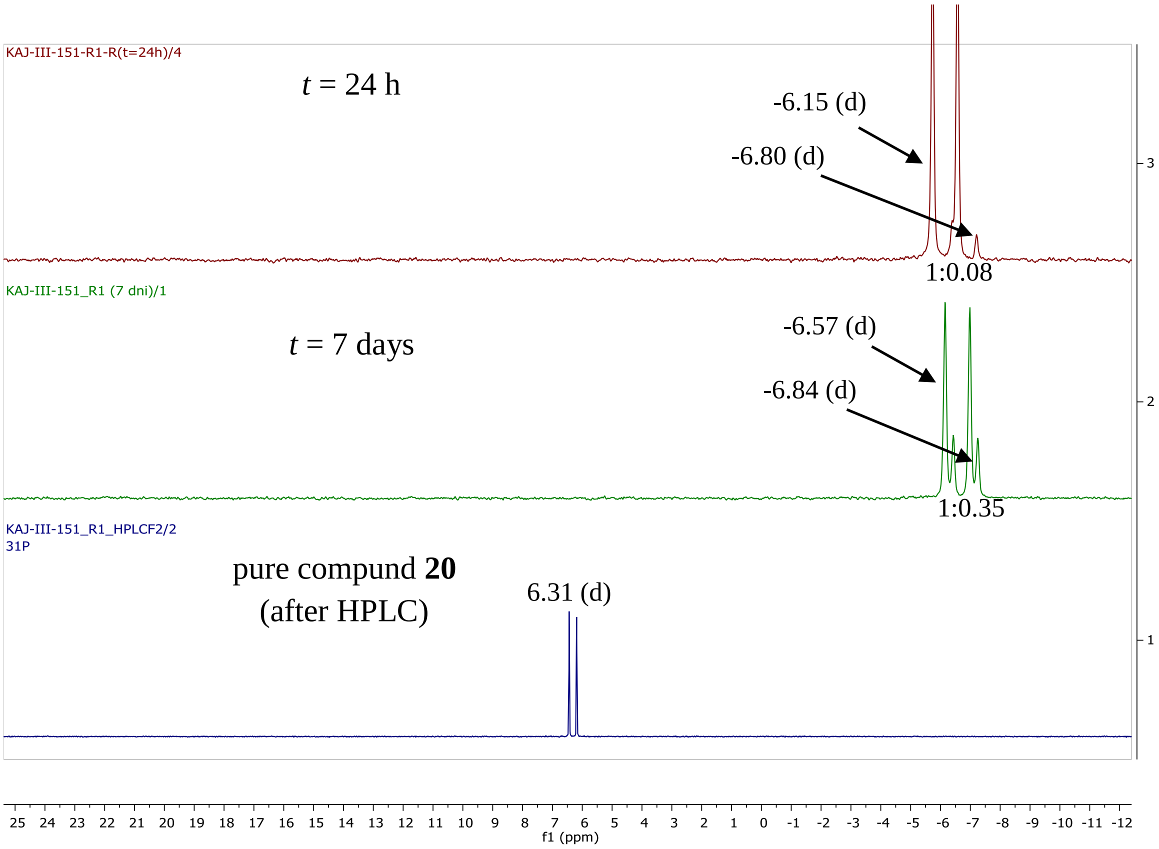

Figure S13: Top and middle: Monitoring of the alkylation reaction progress for compound **9b** at the first stage of the McKenna reaction (BTMS stage); top: ^{31}P NMR after 24 h; middle: ^{31}P NMR after 7 days. Bottom: ^{31}P NMR spectrum of the alkylation product **20** after solvolysis and HPLC purification.

3.5 Compound 22

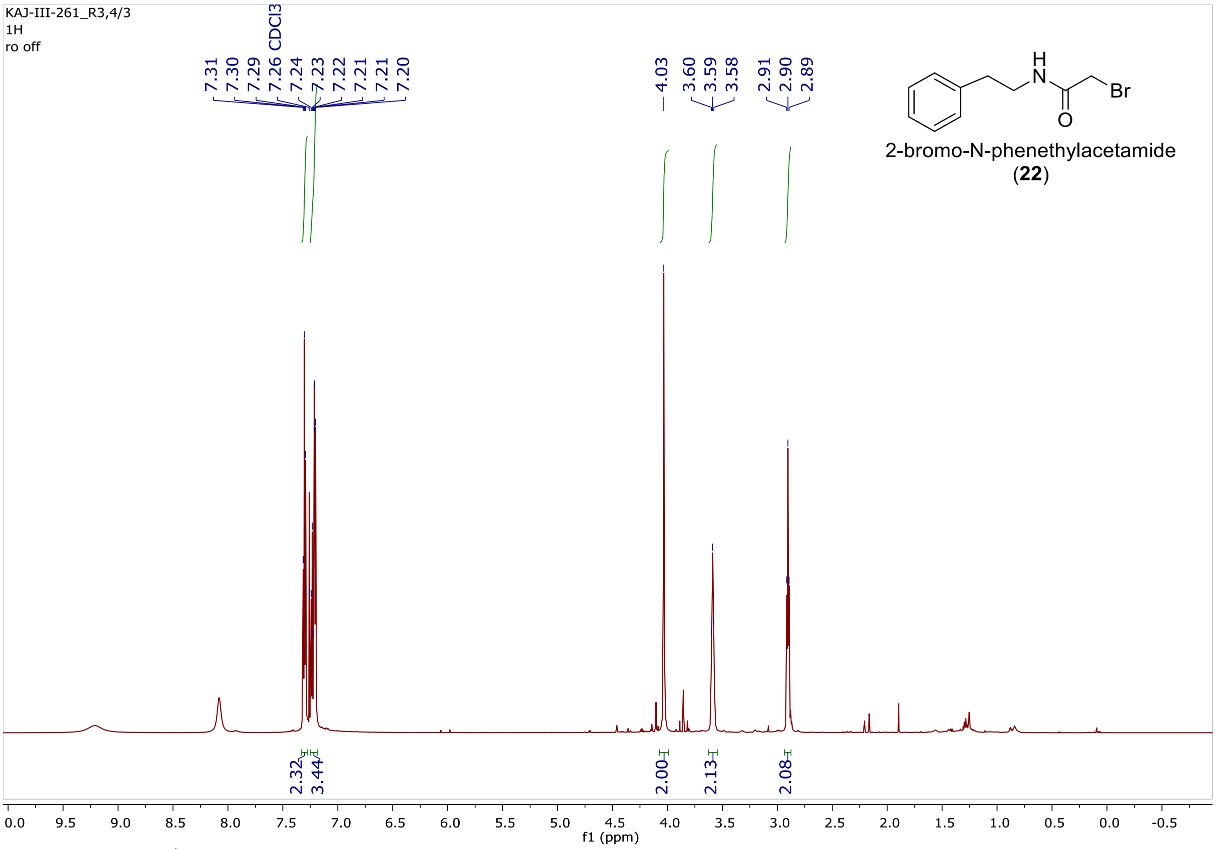

Figure S14: ^{1}H NMR of compound **22** ($CDCl_3$, 700 MHz).

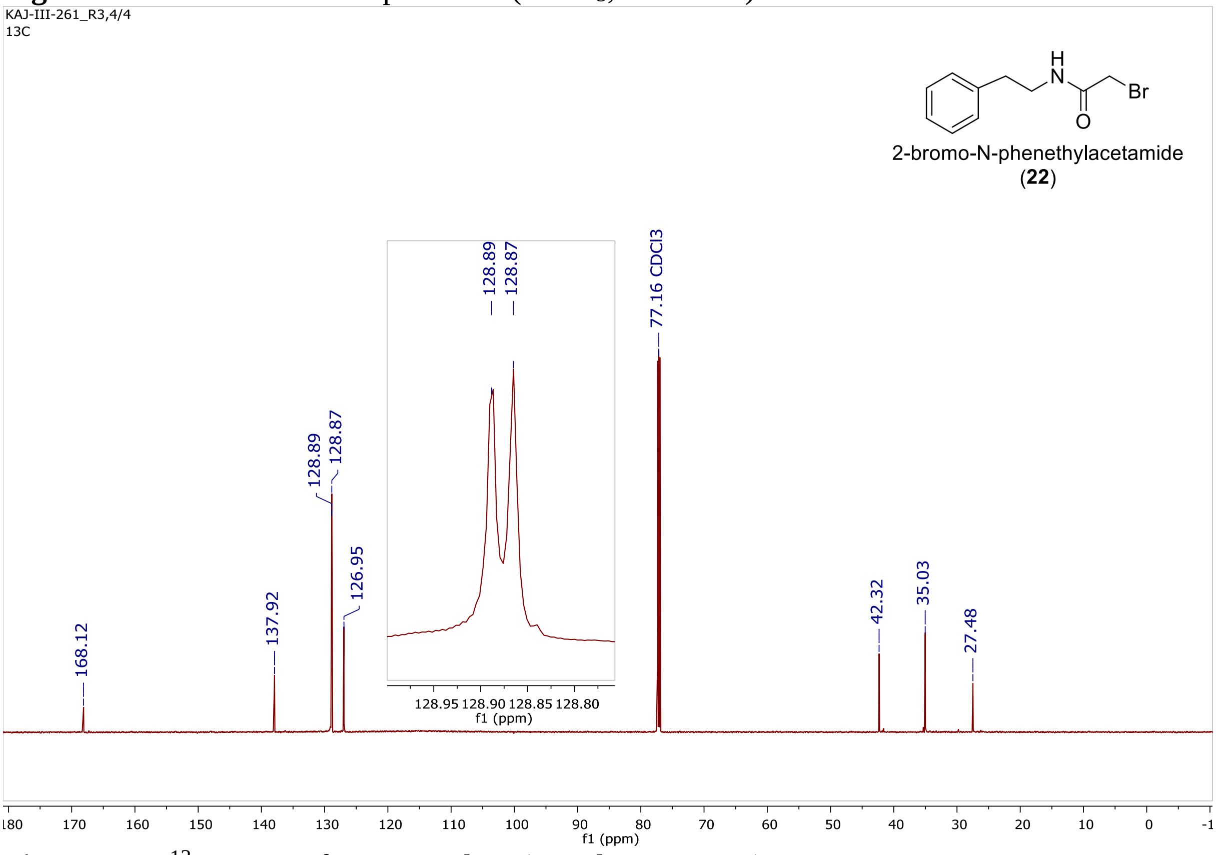

Figure S15: ^{13}C NMR of compound **22** ($CDCl_3$, 176 MHz).

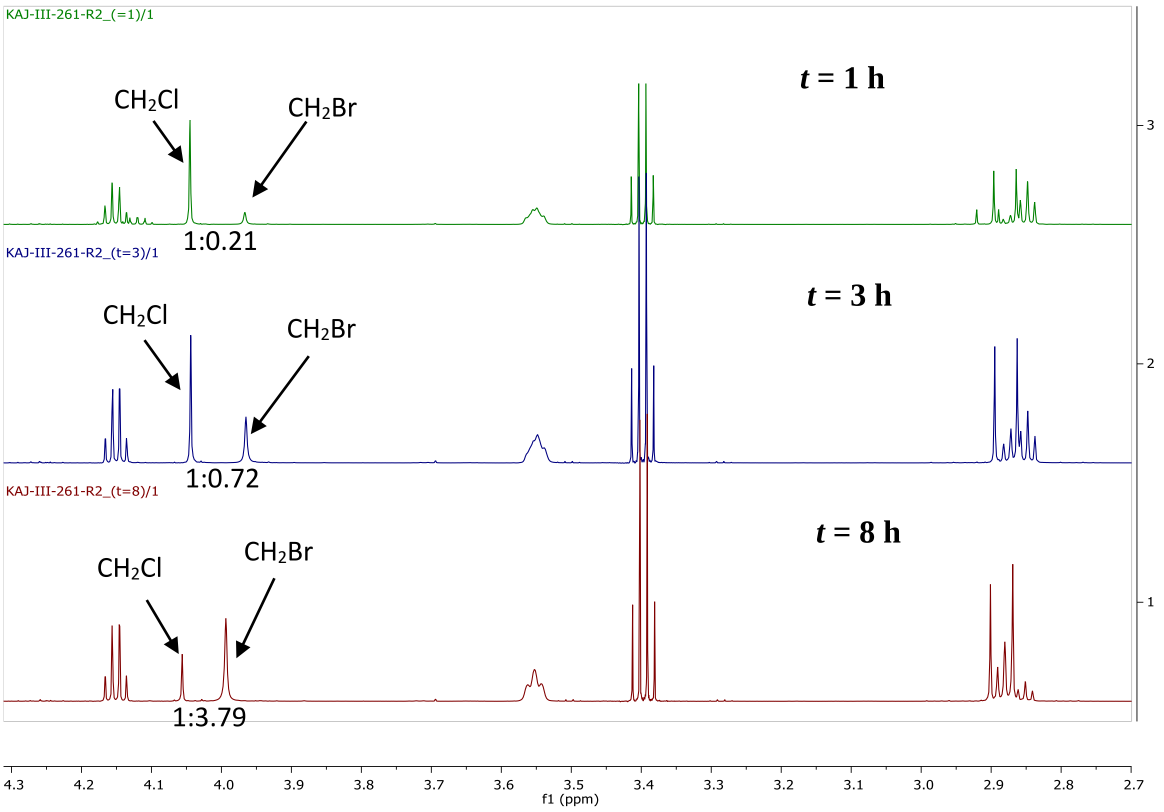

Figure S16: Monitoring by ^{1}H NMR of the reaction progress between **13** and BTMS (CD_3CN, 700 MHz): zoom in of the ranges 2.7–4.3 ppm.

3.6 Compound 21

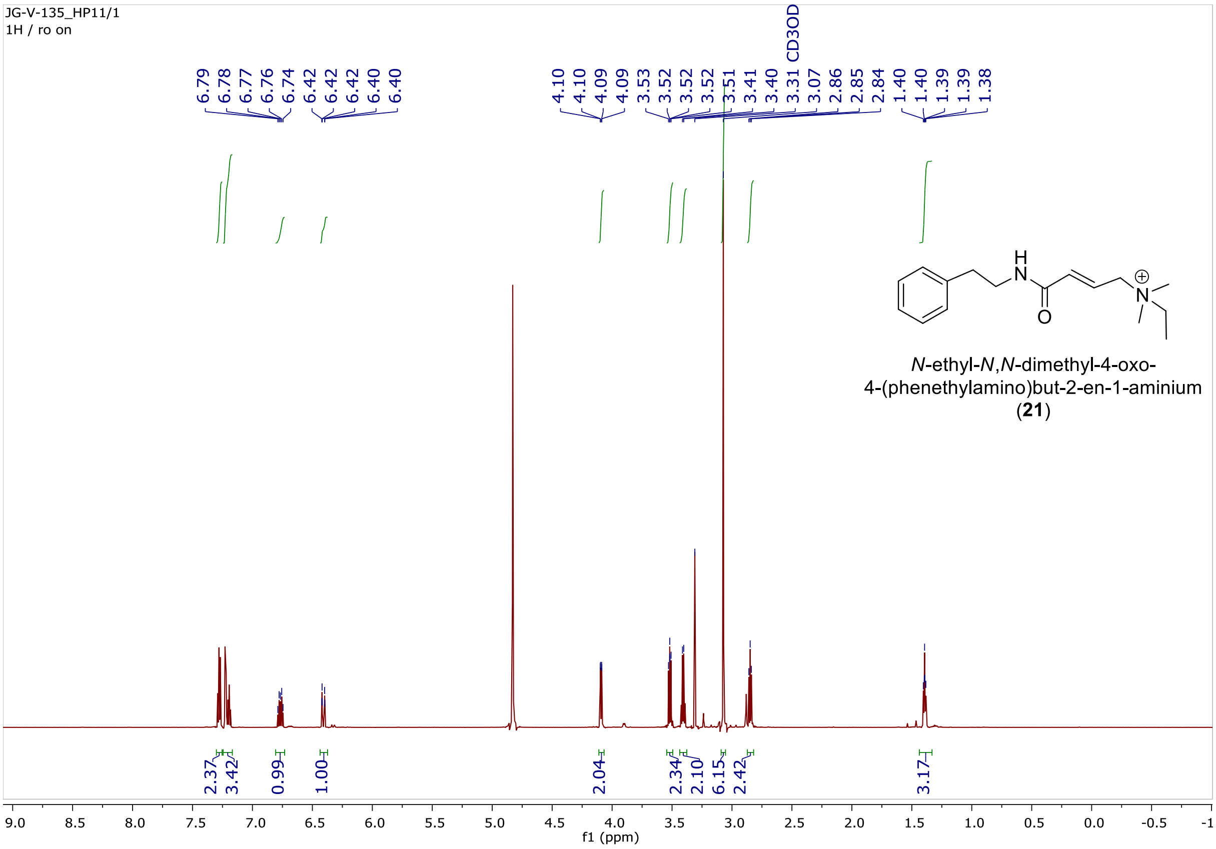

Figure S17: ^{1}H NMR of compound **21** (CD_3OD, 700 MHz).

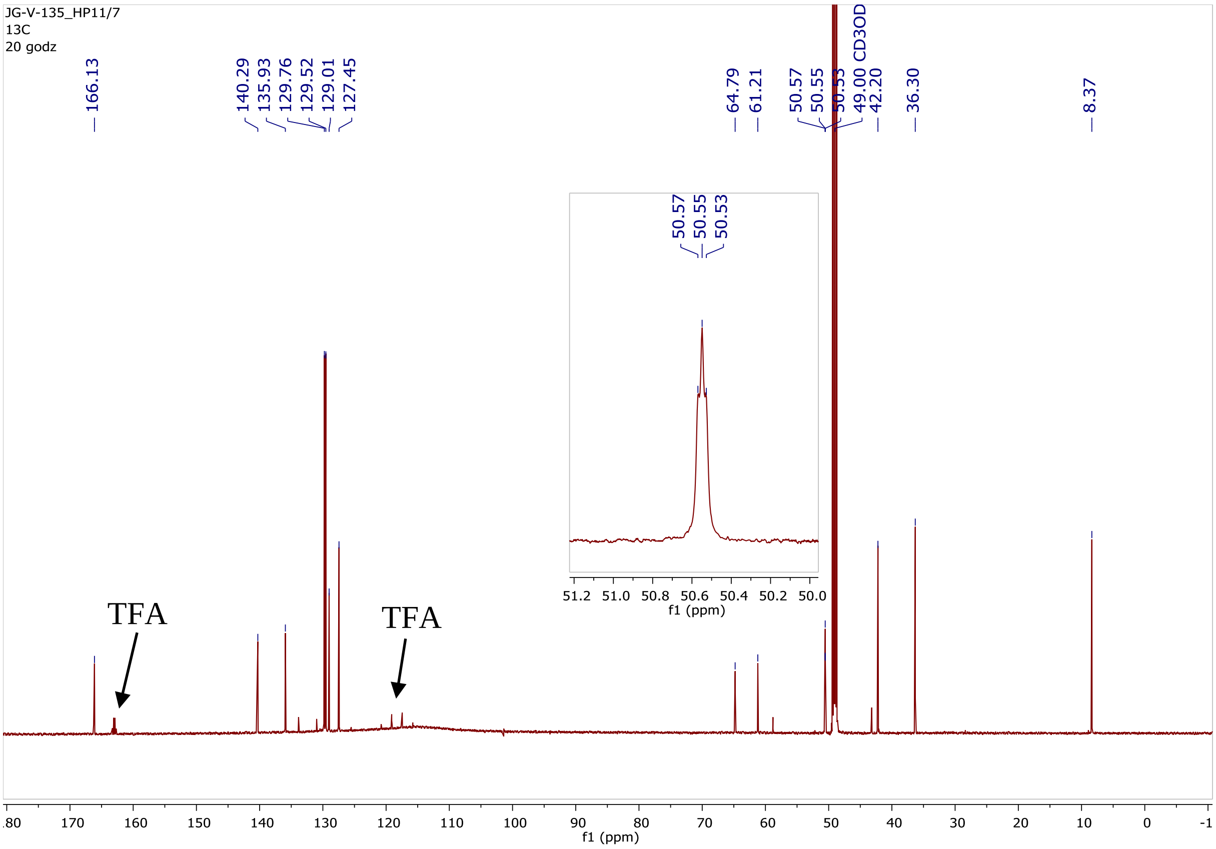

Figure S18: ^{13}C NMR of compound **21** (CD_3OD, 176 MHz).
Note: Compound **21** was purified by HPLC using : A: 95% H_2O + 5% ACN + 0.2% TFA; B: 95% ACN + 5% H_2O + 0.2% TFA, gradient. The peaks of residual TFA were identified in the ^{13}C NMR spectrum.

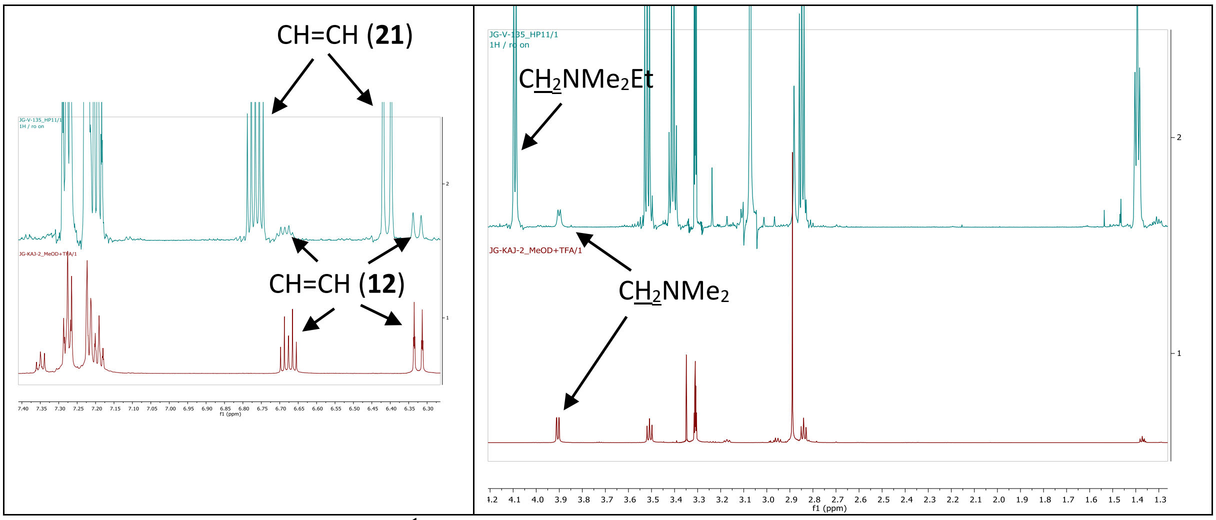

Figure S19: Comparison of the ^1H NMR spectra of substrate **12** (bottom spectrum) and product **21** (top spectrum; compound contaminated with ≈ 10% of substrate **12**) (CD_3OD + TFA, 700 MHz): zoom in of the ranges 1.25–4.20, and 6.30–7.35 ppm. The representative peaks were identified.

3.7 Monitoring the experiments for section 5 by NMR

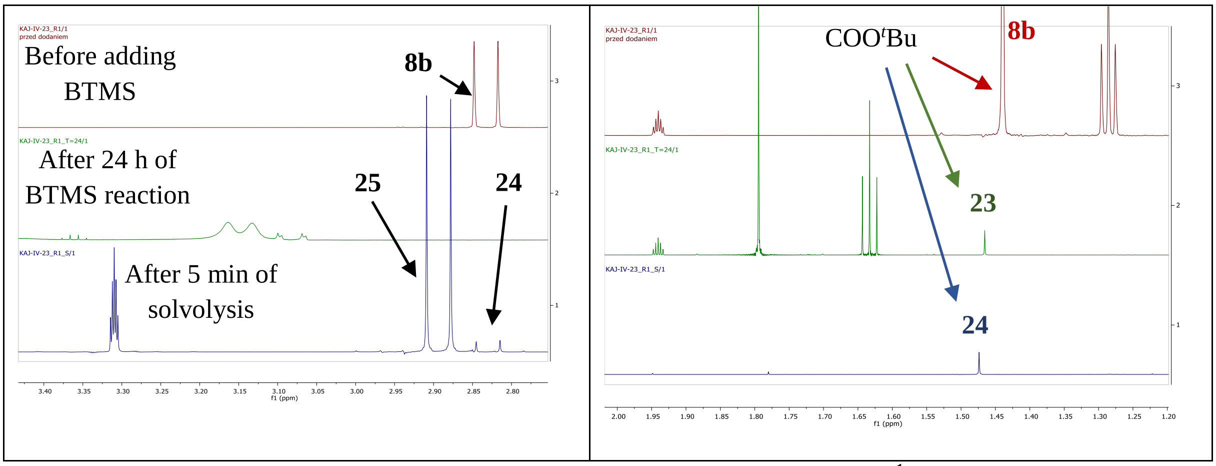

Figure S20: Monitoring of the reaction between **8b** and BTMS: top: ^{1}H NMR spectrum of **8b** before adding BTMS; middle: ^{1}H NMR after 24 h of reaction between **8b** and BTMS (CD_3CN, 700 MHz); bottom: ^{1}H NMR after 5 min of solvolysis (CD_3OD, 700 MHz); zoom in of the ranges 3.45–2.7 and 2.0–1.2 ppm; see Table 4, entry 3.

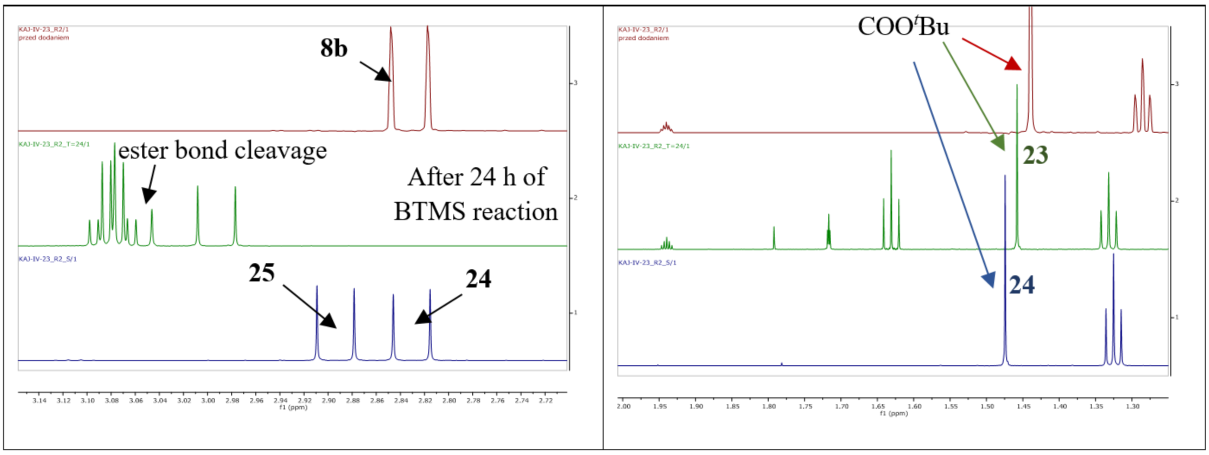

Figure S21: Monitoring of the reaction between **8b** and BTMS in the presence of 1 equiv of TEA: top: ^{1}H NMR spectrum of **8b** before adding BTMS; middle: ^{1}H NMR after 24 h of reaction between **8b** and BTMS (CD_3CN, 700 MHz); bottom: ^{1}H NMR after 5 min of solvolysis (CD_3OD, 700 MHz); zoom in of the ranges 3.15–2.7 and 2.0–1.25 ppm; see Table 4, entry 4.

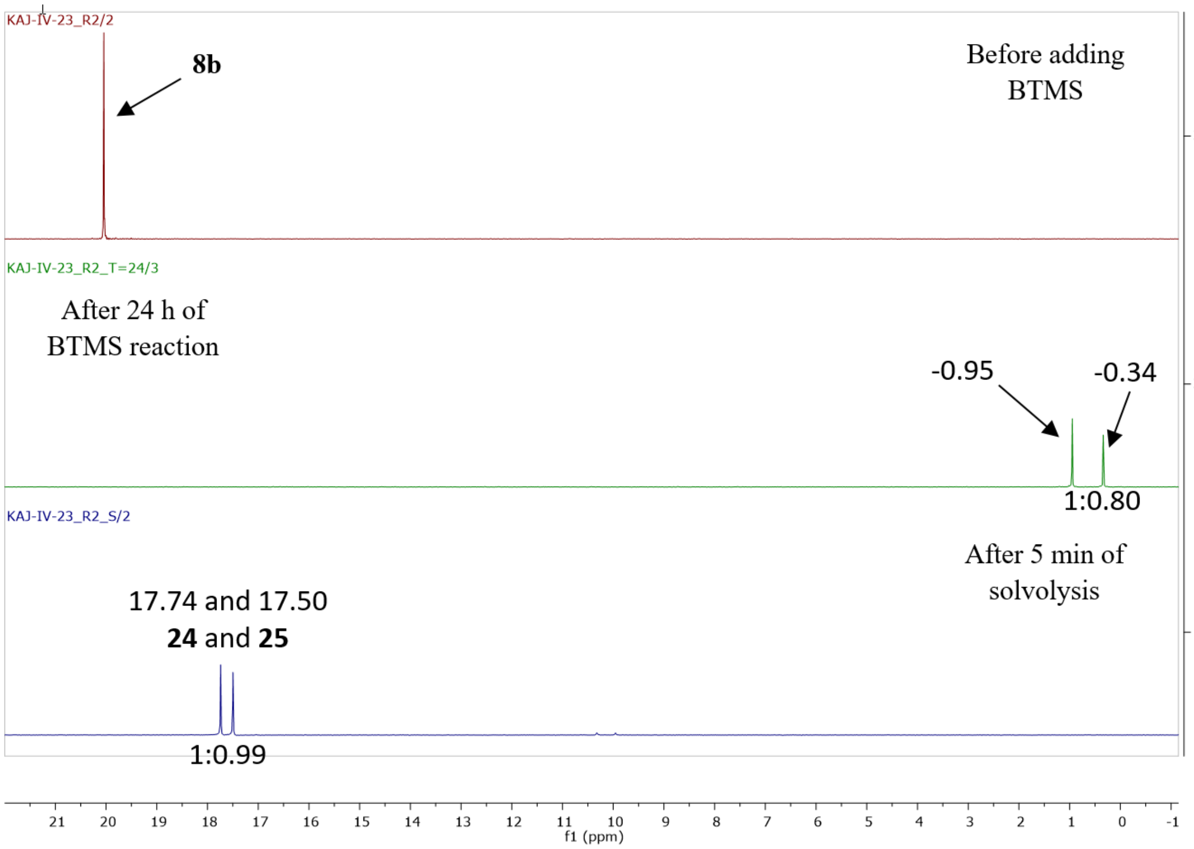

Figure S21a: Monitoring the reaction between **8b** and BTMS in the presence of 1 equiv of TEA: top: ^{31}P NMR spectrum of **8b** before adding BTMS; middle: ^{31}P NMR after 24 h of reaction between **8b** and BTMS (CD_3CN, 700 MHz); bottom: ^{31}P NMR after 5 min of solvolysis (CD_3OD, 700 MHz); zoom in of the ranges 3.15–2.7 and 2.0–1.25 ppm; see Table 4, entry 4.

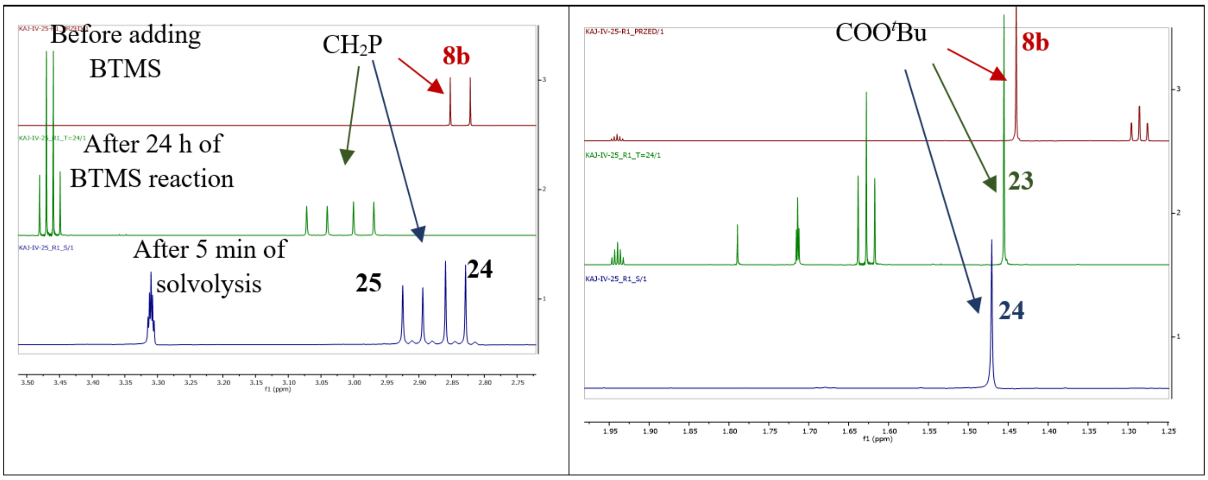

Figure S22: Monitoring the reaction between **8b** and BTMS in the presence of 2 equiv of pyridine: top: ^{1}H NMR spectrum of **8b** before adding of BTMS; middle: ^{1}H NMR after 24 h of reaction between **8b** and BTMS (CD_3CN, 700 MHz); bottom: ^{1}H NMR after 5 min of solvolysis (CD_3OD, 700 MHz); zoom in of the ranges 3.5–2.7 and 2.0–1.25 ppm; see Table 4, entry 5.

4. References

[1] Jiang, C.-s.; Wang, X.-m.; Zhang, S.-q.; Meng, L.-s.; Zhu, W.-h.; Xu, J.; Lu, S.-m. *Bioorg. Med. Chem.* **2015**, *23*, 6510-6519.

[2] Bateman, L. A.; Nguyen, T. B.; Roberts, A. M.; Miyamoto, D. K.; Ku, W. M.; Huffman, T. R.; Petri, Y.; Heslin, M. J.; Contreras, C. M.; Skibola, C. F.; Olzmann, J. A.; Nomura, D. K. *Chem. Commun.* **2017**, *53*, 7234-7237.

[3] Kaźmierczak, A.; Kusy, D.; Niinivehmas, S. P.; Gmach, J.; Joachimiak, Ł.; Pentikäinen, O. T.; Gendaszewska-Darmach, E.; Błażewska, K. M. *J. Med. Chem.* **2017**, *60*, 8781-8800.

[4] Marma, M. S.; Xia, Z.; Stewart, C.; Coxon, F.; Dunford, J. E.; Baron, R.; Kashemirov, B. A.; Ebetino, F. H.; Triffitt, J. T.; Russell, R. G. G.; McKenna, C. E. *J. Med. Chem.* **2007**, *50*, 5967-5975.

[5] Cumine, F.; Zhou, S.; Tuttle, T.; Murphy, J. A. *Org. Biomol. Chem.* **2017**, *15*, 3324-3336.

[6] Safrygin A., Dar'in D., Lukin A., Bakholdina A., Sapegin A., Krasavin M. *Tetrahedron Letters* **2019**, 60, 777–779.

[7] Bodor, N.; Abdel-Alim, A. A. M. *J. Pharm. Sci.* **1985**, *74*, 241-245.

[8] Xie, H.; Ng, D.; Savinov, S. N.; Dey, B.; Kwong, P. D.; Wyatt, R.; Smith, A. B.; Hendrickson, W. A. *J. Med. Chem.* **2007**, *50*, 4898-4908.

[9] APEX-II, Bruker AXS, Madison, Wisconsin, USA.

[10] SAINT, Bruker AXS, Madison, Wisconsin, USA.

[11] SADABS, Bruker AXS, Madison, Wisconsin, USA.

[12] Sheldrick, G. M. (2015). *Acta Cryst.* A**71**, 3-8.

[13] Sheldrick, G. M. (2015). *Acta Cryst.* C**71**, 3-8.

[14] SHELXTL, Bruker AXS, Madison, Wisconsin, USA, 2015.

[15] Bodor N., AbdelAlim A. M. *J. Pharm. Sci.* **1985**, 74, 241-245.
